# TPP-related mitochondrial targeting copper (II) complex induces p53-dependent apoptosis in hepatoma cells through ROS-mediated activation of Drp1

**DOI:** 10.1186/s12964-019-0468-6

**Published:** 2019-11-19

**Authors:** Jiangjuan Shao, Mengmeng Li, Zijian Guo, Chun Jin, Feng Zhang, Chunyan Ou, Yaochen Xie, Shanzhong Tan, Zhenyi Wang, Shizhong Zheng, Xiaoyong Wang

**Affiliations:** 1 Jiangsu Key Laboratory for Pharmacology and Safety Evaluation of Chinese Materia Medica, School of Pharmacy, Nanjing University of Chinese Medicine, Nanjing, 210023 China; 2 State Key Laboratory of Coordination Chemistry, School of Chemistry and Chemical Engineering, Nanjing University, Nanjing, 210023 China; 3 State Key Laboratory of Pharmaceutical Biotechnology, School of Life Sciences, Nanjing University, Nanjing, 210023 China; 4 The Nanjing Hospital Affiliated to Nanjing University of Chinese Medicine, Nanjing, 210003 China; 5 Department of Pharmaceutical Technology, Xuzhou Pharmaceutical Vocational College, Xuzhou, 221116 China

**Keywords:** Copper complex, Hepatocellular carcinoma, Mitochondria, ROS, Drp1, p53, Apoptosis

## Abstract

**Background:**

In recent years, copper complexes have gradually become the focus of potential anticancer drugs due to their available redox properties and low toxicity. In this study, a novel mitochondrion-targeting copper (II) complex, [Cu (ttpy-tpp)Br_2_] Br (simplified as CTB), is first synthesized by our group. CTB with tri-phenyl-phosphine (TPP), a targeting and lipophilic group, can cross the cytoplasmic and mitochondrial membranes of tumor cells. The present study aims to investigate how CTB affects mitochondrial functions and exerts its anti-tumor activity in hepatoma cells.

**Methods:**

Multiple molecular experiments including Flow cytometry, Western blot, Immunofluorescence, Tracker staining, Transmission Electron Microscopy and Molecular docking simulation were used to elucidate the underlying mechanisms. Human hepatoma cells were subcutaneously injected into right armpit of male nude mice for evaluating the effects of CTB in vivo.

**Results:**

CTB induced apoptosis via collapse of mitochondrial membrane potential (MMP), ROS production, Bax mitochondrial aggregation as well as cytochrome c release, indicating that CTB-induced apoptosis was associated with mitochondrial pathway in human hepatoma cells. Mechanistic study revealed that ROS-related mitochondrial translocation of p53 was involved in CTB-mediated apoptosis. Simultaneously, elevated mitochondrial Drp1 levels were also observed, and interruption of Drp1 activation played critical role in p53-dependent apoptosis. CTB also strongly suppressed the growth of liver cancer xenografts in vivo.

**Conclusion:**

In human hepatoma cells, CTB primarily induces mitochondrial dysfunction and promotes accumulation of ROS, leading to activation of Drp1. These stimulation signals accelerate mitochondrial accumulation of p53 and lead to the eventual apoptosis. Our research shows that CTB merits further evaluation as a chemotherapeutic agent for the treatment of Hepatocellular carcinoma (HCC).

## Background

Hepatocellular carcinoma (HCC) is one of the aggressive types of tumor spread worldwide, which is originated chiefly from chronic liver diseases [1]. To date, although remarkable progress has been achieved in conventional treatment, it remains the most lethal malignancies globally due to limited restricted therapeutics, high recurrence rate and poor prognosis [2, 3]. Due to the remarkable efficacy of metal drugs in the treatment of various cancers, the study of metal complexes has long been a hot topic [4, 5]. The metals involved in the antitumor complexes mainly include platinum-based anticancer drugs, such as cisplatin, carboplatin, and oxaliplatin [6, 7]. However, the lack of selectivity leads to the occurrence of side effects such as drug resistance, and hence their application has been greatly limited. The physiological distribution and intracellular accumulation of copper complexes differ greatly from platinum complexes, which bring prospects for copper complexes as antitumor drugs to overcome drug resistance [8, 9].

Studies on copper complexes have shown significant progresses in the antitumor activity, while the study on copper complexes with targeting groups to organelles of the cell is rare. Cancer cells have higher mitochondrial membrane potentials (ΔΨm), which can be used to deliver drugs selectively to cancer cells whilst bypass normal cells from toxicity [10, 11]. Triphenylphosphine (TPP) has been used as a mitochondrion-targeting moiety to develop drugs in cancer therapy, because it can impart a delocalized charge and lipophilic character to the compound, which is beneficial to mitochondrial accumulation [12–14]. Studies on Cu-labeled triphenylphosphonium cations as imaging agents showed that these copper complexes preferentially accumulate within tumor cells because of the increased negative ΔΨm and have low uptake in the heart and muscle due to their high hydrophilicity [15]. Recently, our group introduced TPP into a metal complex to obtain a new copper complex [Cu (ttpy-tpp)Br_2_] Br (expressed as CTB). This complex has good water solubility, DNA cleavage activity and a significant mitochondrial targeting ability [16]. However, the molecular mechanism by which CTB exerts a strong antitumor effect via targeting mitochondrial pathway in human liver cancer cells has not yet been explored.

Resistance to the mechanism of cell death is characteristic of malignant cells. Accordingly, induction of cell apoptosis is a primary target of anti-tumor therapy. Mitochondria play a critical role in mediating intrinsic pathway of apoptosis in mammalian cells [17, 18]. In response to external stimuli, mitochondrial out membrane permeabilization (MOMP) increases, which is regulated by pro-apoptotic proteins (Bax, Bak) and the anti-apoptotic proteins (Bcl-2, Bcl-xL) [19, 20]. Several drugs and compounds may disrupt the mitochondrial machinery by raising oxidative stress, a process that enhances the production of ROS [21–23]. Accumulation of ROS can lead to loss of MMP and mPTP opening, thus allowing the release of cytochrome c (Cyt C) into the cytoplasm and the initiation of a caspase cascade reaction. Notably, CTB has been shown to induce oxidative stress, leading to DNA damage [16], which urges us to investigate whether CTB can induce hepatoma cell apoptosis.

p53 is a key regulator of survival and proliferation, but other functions were more recently assigned to p53, notably control of metabolism, oxidative stress, DNA repair, angiogenesis, apoptosis, senescence and autophagy [24]. p53 exerts dual effects on oxidative stress: protection at basal levels and apoptosis at high ROS levels, which were directly linked to its antitumor effects. Recent studies also suggested that once subjected to some kind of stimulation, p53 may activate cell apoptosis in a transcription-independent manner and interact with Bcl-2 family members in mitochondria [25, 26]. Natsumi Noda developed a bioluminescent probe to monitor p53 translocation from cytosol to mitochondria using luciferase fragment complementation assays [27]. However, the specific mechanism by which p53 transferring to mitochondria remains unclear. As dynamic organelles, mitochondria continuously undergo fusion and fission to adapt to changing conditions [28]. Mitochondrial fission is mainly controlled by Dynamin-related protein 1 (Drp1), regulating cellular mitosis and removal of damaged mitochondria by mitophagy [29, 30]. Drp1 is a member of the GTP enzyme dynamic superfamily, which modulates diverse cellular functions, including vesicle fission, organelle division, resistance to viruses and intracellular trafficking [31, 32]. In response to various stresses, Drp1 aggregates on the mitochondrial outer membrane and interacts with Bax, disturbing the MOMP function and the release of pro-apoptotic factors [33, 34].

In the present study, we investigated how CTB induces apoptosis in HCC cells. We found that CTB had the ability to induce mitochondrial apoptosis in hepatoma cells and inhibited tumor growth in SMMC-7721 xenograft mouse. Mechanistic studies revealed that interruption of the ROS production, the Drp1 activation and mitochondrial p53 played critical role in CTB-mediated opening of mPTP, depletion of ATP and mitochondrial apoptosis ultimately. Our research provides novel insight into apoptotic effects of copper complex and suggests that CTB may be a promising valuable chemotherapeutic agent for the treatment of HCC, thus providing a theoretical basis for the future design of mitochondria-targeted drugs.

## Materials and methods

### Reagents and antibodies

CTB ([Cu (ttpy-tpp)Br_2_]Br) was provided by State Key Laboratory of Coordination Chemistry in Nanjing University. All antibodies were used at a dilution of 1:1000 unless otherwise specified according to the instructions. Antibodies to Cleaved-PARP (#5625), PARP (#9532), Cleaved-caspase-9 (#20750), Cleaved-caspase-3 (#9664), Caspase-9 (#9502), Caspase-3 (#9662), Bax (#14796), Bcl-2 (#15071), Cytochrome c (#12963), p53 (#2557), Mitofusin-1 (#14739), Mitofusin-1 (#9482), Drp1 (#8570), p-DRP1 (Ser616) (#D9A1), β-actin (#3700) and COX IV (#38563) were purchased from Cell Signaling Technology (Danvers, MA, USA). Anti-rabbit IgG was purchased from Sigma-Aldrich (St. Louis, MO, USA). Pifithrin-μ and Mdivi-1 were purchased from Cayman Chemical (Ann Arbor, MI, USA), were dissolved in dimethylsulfoxide (DMSO; Sigma-Aldrich, St. Louis, MO, USA) according to the specification. NAC was purchased from MedChem Express (New Jersey, USA).

### Cells culture

Human hepatocellular carcinoma cells were purchased from the KeyGEN BioTECH (Nanjing, Chinese). Cells were cultured in RPMI 1640 (KeyGEN BioTECH, Nanjing, Chinese) supplemented with 10% fetal bovine serum (FBS; Gibco, Invitrogen, Merelbeke, Belgium), 100 U mL^− 1^ penicillin, and 100 μg mL^− 1^ streptomycin in incubator under the controlled condition of a humidified atmosphere of 95% air and 5% CO_2_ at 37 °C. Cell morphology was observed by Leica Qwin System. Prior to CTB treatment, cells growed to approximately 70–80% confluence and then were exposed to different concentrations (0–4 μM) of CTB for different time periods (0–24 h).

### Animals and experimental procedures

All experimental procedures were approved by the institutional and local committee on the care and use of animals of Nanjing University of Chinese Medicine (Nanjing, China), and all animals received humane care according to the National Institutes of Health (USA) guidelines. Four-week-old male nude mice (BALB/c-nu/nu) weighing approximately 18–22 g were procured from Nanjing Institute of Biomedical Research (Nanjing, China). All mice were housed in cages under germ-free conditions with a 12-h light-dark cycle and sufficient water and food.

To establish human HCC xenograft model, SMMC-7721 cells were harvested at logarithmic phase, and 1 × 10^7^ cells/200 μL were subcutaneously injected into right armpit of each mouse to induce tumor growth. After transplantation, the tumor sizes were measured using calipers, and the tumor volumes were estimated every 3 days. To establish the growth curve, the long diameter (a) and short diameter (b) were measured by Vernier caliper and the tumor volumes were calculated by the formula which is volume (V) = a × b^2^/2.When the tumors had reached a mean size of 150 mm^3^, the mice were randomly divided into seven groups (6 animals per group). Mice of Group 1 were served as a subcutaneous xenograft model. Mice of Group 2 were served as a positive control group and i.p. injected by Cis-Pt with 10 mg kg^− 1^. Mice of Groups 3, 4 and 5 were served as treatment groups and i.p. injected by CTB with 2.5, 5 and 10 mg kg^− 1^, respectively. Mice of Group 6 were i.p. injected by Pifithrin-μ (8 mg kg^− 1^). Mice of Group 7 were served as a combined administration group and i.p. injected by CTB (5 mg kg^− 1^) and Pifithrin-μ (8 mg kg^− 1^). CTB was suspended in sterile PBS and injected three times a week, and the model group received the same volume of saline. Body weight was recorded every 3 days. After mice were sacrificed, their tumors were photographed and weighed to calculate the tumor inhibition rate. Inhibition rate (%) = (average tumor weight in control group – average tumor weight in treatment group) / average tumor weight in control group × 100%. Excision of parts of the tumor tissue were fixed in 4% paraformaldehyde for IHC assay or frozen in liquid nitrogen.

### MTT assay

The cells were seeded in 96-well plates and cultured for 12 h in medium supplemented with 10% FBS and then treated with the indicated dose of CTB for an additional 24 h. Five duplicate wells were set up for each group. Thereafter, 3-(4,5-dimethylthiazol-2-yl)-2,5- diphenyltetrazolium bromide (MTT, 20 μL, 5 mg mL^− 1^; Biosharp, Nanjing, China) was added to each well of a 96-well plate, followed by incubation for an additional 4 h. Subsequently, DMSO (200 μL) was added to each well to dilute the solid matter in each well. Absorbance values at 490 nm were obtained by using a SpectraMax™ microplate spectrophotometer (Molecular Devices, Sunnyvale, CA).

### Biochemical analysis

Commercial assay kits detected a series of hepatocyte injury indicators, such as AST, ALT and LDH, according to the protocols from the manufacturer (Nanjing Jiancheng Bioengineering Institute, Nanjing, China). Absorbance values were obtained by using a SpectraMaxTM microplate spectrophotometer (Molecular Devices, Sunnyvale, CA) and the relative concentrations were calculated.

### Western blot analysis

Cells or tissue samples were lysed using mammalian lysis buffer (Sigma, St. Louis, MO, USA) and immunoblotting was performed as we described previously. Meanwhile, BCA assay kit (Beyotime, Jiangsu, China) measures the concentration of protein obtained. Proteins (50 mg per well) were separated by sodium dodecyl sulfate-polyacrylamide gel, then transferred to a polyvinylidene fluoride membrane (IPVH00010, Millipore, Burlington, MA, USA), blocked with 5% skim milk in Tris-buffered saline containing 0.1% Tween 20 for 2 h. The target protein was detected by the corresponding primary antibody and secondary antibody. Protein bands were visualized using a luminescent liquid (Millipore). β-actin was used as an invariant control for total protein and cytoplasmic proteins, and COX IV was used for mitochondrial proteins. The levels of target protein were densitometrically determined using Image Lab.

### Transmission electron microscopy analysis

Cells were seeded onto 4-chambered coverglass (Lab-Tek Chambered Coverglass System) (Nalgene/Nunc, Rochester, NY, USA) at a density of 2 × 10^4^ cells/ml (14,000 cells/well). Images were acquired using the Olympus EM208S transmission electron microscope.

### Immunofluorescence analysis

To assess the subcellular localization of p53/Drp1, we used laser confocal imaging of cells double labeled with MitoGreen probe and p53/Drp1 primary antibody. SMMC-7721 cells were incubated with MitoGreen (200 nM) probe in an incubator for 30 min prior to treatment. Next, the cells were washed with PBS and fixed with 4% paraformaldehyde at 37 °C for 30 min. The fixed cells were permeabilized with 0.1% Triton X-100 for 10 min at room temperature. The cells were then incubated for 1 h in blocking solution (1% BSA in PBS) and primary monoclonal p53/Drp1 antibody (1:500) was incubated for 4 h at room temperature. After washing, cells were incubated for 2 h in 1% BSA containing TRITC-conjugated goat anti-rabbit antibody (1:200). After staining with DAPI (5 μg mL^− 1^) for 10 min at 37 °C, images were obtained on a confocal microscope. Mito-tracker had excitation and emission wavelengths of 490/516 nm; for TRITC-conjugates, they were 550/570 nm, respectively, and all images were captured with a fluorescence microscope and representative images were displayed.

### Apoptosis detection

SMMC-7721 cells were harvested and suspended in 500 μL of binding buffer, which was mixed with FITC-labeled annexin-V (10 μL) and PI (10 μL). The cells were incubated for 30 min at room temperature and then detected by an Accuri C6 flow cytometer (BD Sciences, USA). The scattering parameters of the cells (1 × 10^4^ cells) were analyzed using the Flow plus system. Apoptosis was also assessed by the TUNEL Apoptosis Detection Kit. After the treatment, the cells were observed under a fluorescence microscope (Nikon, Tokyo, Japan).

### ICP for Cu^2+^ concentration detection

Tumor cells were cultured in RPMI-1640 (containing 10% FBS) and complex (2 μM) for 24 h at 5% CO_2_ and 37 °C. The mitochondria in the cells were gradually extracted through the mitochondrial extraction kit in accordance with the procedures. The extracted mitochondria were dissolved in 1 mL of Milli-Q and the Cu^2+^ content was determined by ICP-MS. At the same time, another group of cells under the same culture conditions were sequentially treated with Milli-Q (50 μL), concentrated nitric acid (70 μL, 95 °C, 2 h), 30% hydrogen peroxide (20 μL, 95 °C, 1.5 h). Concentrated hydrochloric acid (35 μL) was digested, and the digested cell fluid was diluted to 1 mL with Milli-Q. The Cu^2+^ content was determined by ICP-MS.

### Reactive oxygen species (ROS)measurement

SMMC-7721 cells were cultured in a 24-well plate at a density of 1 × 10^5^ cells/well for 12 h and then treated with CTB (1, 2 or 4 μM) for 24 h. The ROS detection kit (Beyotime, Jiangsu, China) was used to visualize intracellular ROS fluorescence using a fluorescent probe DCFH-DA under a fluorescence microscope (Nikon, Tokyo, Japan).

### Mitochondrial membrane potential (ΔΨm) measurement

The mitochondrion-specifc lipophilic cationic fluorescence dye JC-1 (Beyotime, Jiangsu, China) was commonly used to detect mitochondrial membrane potential. SMMC-7721 cells were cultured in 24-well plates (2 × 10^5^/well) and incubated at 37 °C for 24 h. Next, the cells were treated with different concentrations of CTB for 24 h, after which they were collected and incubated with 10 mM JC-1 in the dark at 37 °C for 30 min and were washed twice with PBS. Taking photographs used a fluorescence microscope (Nikon, Tokyo, Japan).

### Rhodamine 123 staining

Rhodamine 123 is a cationic fluorescent dye that can penetrate cell membranes. When the integrity of the mitochondrial membrane is destroyed, Rh123 re-releases the mitochondria, thereby emitting strong fluorescence. The cells were cultured in 24-well plates (1 × 10^6^) after drug treatment. Rhodamine 123 staining solution (Beyotime, Jiangsu, China) (10 μg mL^− 1^) was added, and incubation was carried out for 30 min at 37 °C in a 5% CO_2_ cell incubator. Laser microscopy detection: excitation wavelength 488–505 nm, emission wavelength 515–575 nm.

### Mitochondrial protein extraction

Using Mitochondria/Cytosolic Fractionation Kit (Jiancheng Bioengineering Institute, Nanjing, China) separated and extracted mitochondria in cultured cells and tumor tissues. SMMC-7721 cells or tumor tissues were homogenized in an ice bath and centrifuged at 2000 g for 5 min, approximately 5000 cells needed for each extraction. The mixture was re-suspended in PBS, and the cell suspension is transferred to a small volume of glass homogenate for grinding. Then the cell or tissue homogenate were centrifuged at 8000 g for 5 min. The Supernatant were collected as intact mitochondria. In order to collect mitochondrial protein, the purer isolated mitochondria were added to the lysate for lysis and extraction.

### ATP level determination

The ATP Assay Kit (Beyotime, Jiangsu, China) was used to measure ATP production levels in SMMC-7721 cells. The prepared cells were homogenized with the lysis buffer and centrifuged at 12000 g for 10 min, collecting the supernatant for subsequent determination. Twenty μL samples were mixed with 180 μL reacting buffer. Luminescence was monitored at 560 nm using luminometer. In order to eliminate the error caused by the difference in protein amount during sample preparation, the protein concentration in the sample can be determined. Finally, the concentration of ATP was converted into the form of nmol mg^− 1^ protein.

### SOD enzyme activity

The MnSOD (SOD2) activity detection kit (WST-8) (Beyotime, Jiangsu, China) detected the production of mitochondrial SOD in cells and tissues by colorimetry. The cells treated with CTB were collected and washed 2 times with PBS precooled at 4 °C or ice bath. Thereafter, the mixture was homogenized in an ice bath with pre-cooled PBS, and then the supernatant was taken as a sample to be tested. Using a 96-well plate, sample wells and blank control groups were set. The absorbance was measured at 450/405 nm.

### Statistical analysis

All the quantitative data were described as the mean ± standard deviation (SD) of at least three independent experiments and were analyzed using GraphPad Prism 5 (San Diego, CA, USA). The significance of difference was determined by one-way ANOVA with the post-hoc Dunnett’s test. Values of *P* < 0.05 were considered to be statistically significant.

## Results

### CTB inhibits cell proliferation and induces cell cycle arrest in HCC

**Fig. 1:**
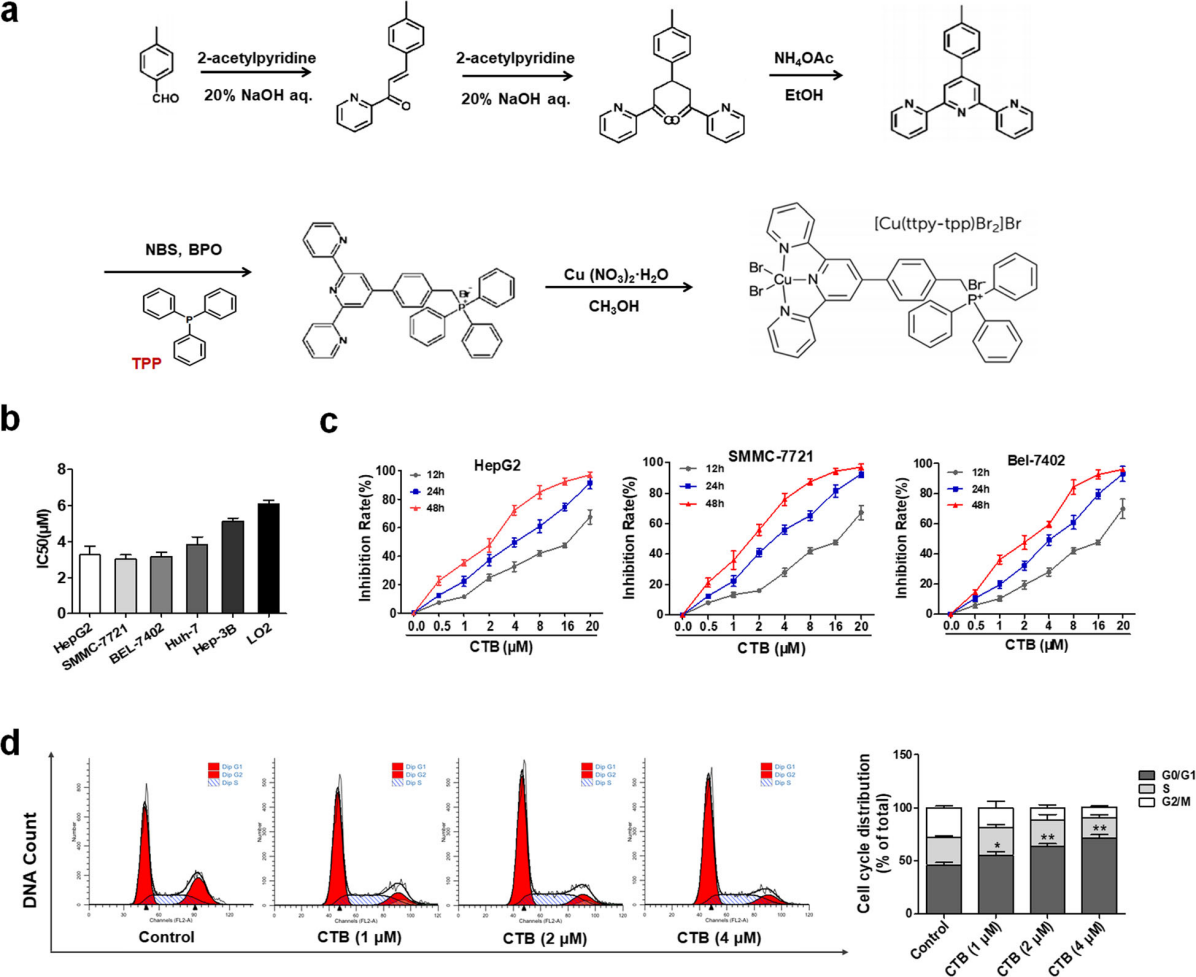
CTB inhibits cell proliferation and induces cell cycle arrest in HCC. **a** Chemical structure and molecular formula of CTB. **b** HepG2, SMMC-7721, BEL-7402, Huh-7, Hep3B and LO2 cells were treated with CTB at different concentrations for 24 h, and the IC50 values were quantified using the MTT assay. **c** HepG2, SMMC-7721, BEL-7402 cells were treated with different CTB concentrations (0, 0.5, 1, 2, 4, 8, 16 or 20 μM) for 12, 24, 48 h, and cell viability was determined by the MTT assay. **d** Cell cycle analysis by flow cytometry. Percentages of cell cycle distributions were determined. Data are represented as the mean ± SD (*n* = 3). *P < 0.05, **P < 0.01 and ***P < 0.001 vs Control

As shown in Fig. 1a, the tripyridine ligand, 4′-p-tolyl-2,2′:6,2″-terpyridine (ttpy), was synthesized first, and then TPP was introduced into ttpy-tpp, coordinating with copper (II) to obtain CTB. The copper-terpyridine complex preferentially accumulates within tumor cells due to their nuclease activity and DNA cleavage activit y[16]. TPP confers delocalized charge and lipophilic properties on the compound, favoring mitochondria accumulation. Firstly, the cytotoxicity of CTB on human liver cancer cells (HepG2, SMMC-7721, BEL-7402, Huh-7, Hep3B) and human hepatocytes (LO2) at 24 h was tested by MTT analysis, showing that LO2 cells were less sensitive to CTB since the IC50 at 24 h was higher than that of other hepatoma cells respectively (Fig. 1b). At the same time, SMMC-7721 is most sensitive to CTB compared to other hepatoma cell lines. HepG2, SMMC-7721 and BEL-7402 cells were treated with different CTB concentrations (0, 0.5, 1, 2, 4, 8, 16 or 20 μM) for the indicated times, and cell proliferation was determined. Exposure to CTB, the proliferation of hepatoma cells was significantly inhibited in a dose- and time-dependent manner (Fig. 1c). The cell viability of hepatocytes was also tested to further determine the dose of CTB (Additional file 1: Figure S1A, B). In addition, CTB caused abnormal high activities of ALT, AST and LDH in serum in cell supernatant following exposure to CTB (> 4 μM) (Additional file 1: Figure S1C). Inhibition of growth and proliferation of hepatoma cells is inseparable from regulation of cell cycle. SMMC-7721 cells exposure to CTB (0, 1, 2, 4 μM) for 24 h, flow cytometric analysis showed that G0/G1 cell cycle arrest was induced by CTB in a dose-dependent manner (Fig. 1d). Under the signal of G0/G1 cell cycle arrest, what kind of death program is initiated by hepatoma cells still needs further study. However, it can also be preliminarily indicated that CTB-inhibited proliferation of hepatoma cells is associated with cell cycle arrest.

### The confirmation of mitochondrial targeting characteristic of CTB in hepatoma cells

In sporadic examples, selected ligands are used to direct the toxicity of the metal toward tumor cells but ligands with properties targeting a specific cellular organelle are rarely seen in copper complexes. Based on the above findings, whether the cause of CTB exerts anti-tumor activity is related to its mitochondrial targeting characteristics needs further confirmation. Firstly, we identified the mitochondrial aggregation characteristics of CTB in SMMC-7721 cells. The contents of copper ions of complexes, Cu[(ttpy-tpp)Br_2_] Br (CTB) and Cu (ttpy)Br_2_ (no TPP), in SMMC-7721 cells and mitochondria were detected by ICP-MS. The specific data (Table 1) indicated that two complexes can enter the cell through the cell membrane. Cu[(ttpy-tpp)Br_2_] Br had a density of 3.32 ng/μL in the mitochondria, which was higher than the density of 3.09 ng/μL in the whole cell. The density of Cu (ttpy)Br_2_ in the mitochondria was 1.08 ng/μL, which was lower than the density of 3.37 ng/μL in the whole cell. Thus, the introduction of the mitochondrial targeting group TPP in CTB facilitates the entry of the complex into the mitochondria.

**Table 1:**
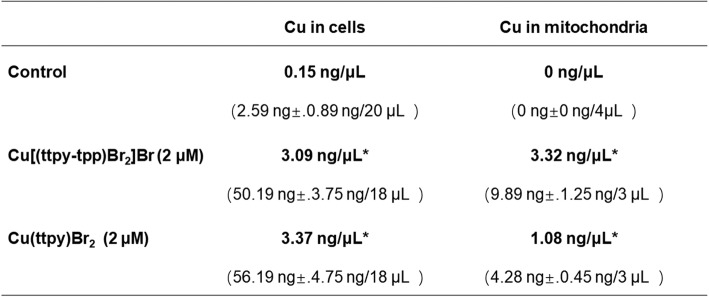
The copper ions of Cu[(ttpy-tpp)Br_2_] Br and Cu (ttpy)Br_2_ enter the mass and density of each 10^6^ SMMC-7721 cells and their mitochondria (average of three parallel experiments. *P < 0.05 compared with control group

**Fig. 2:**
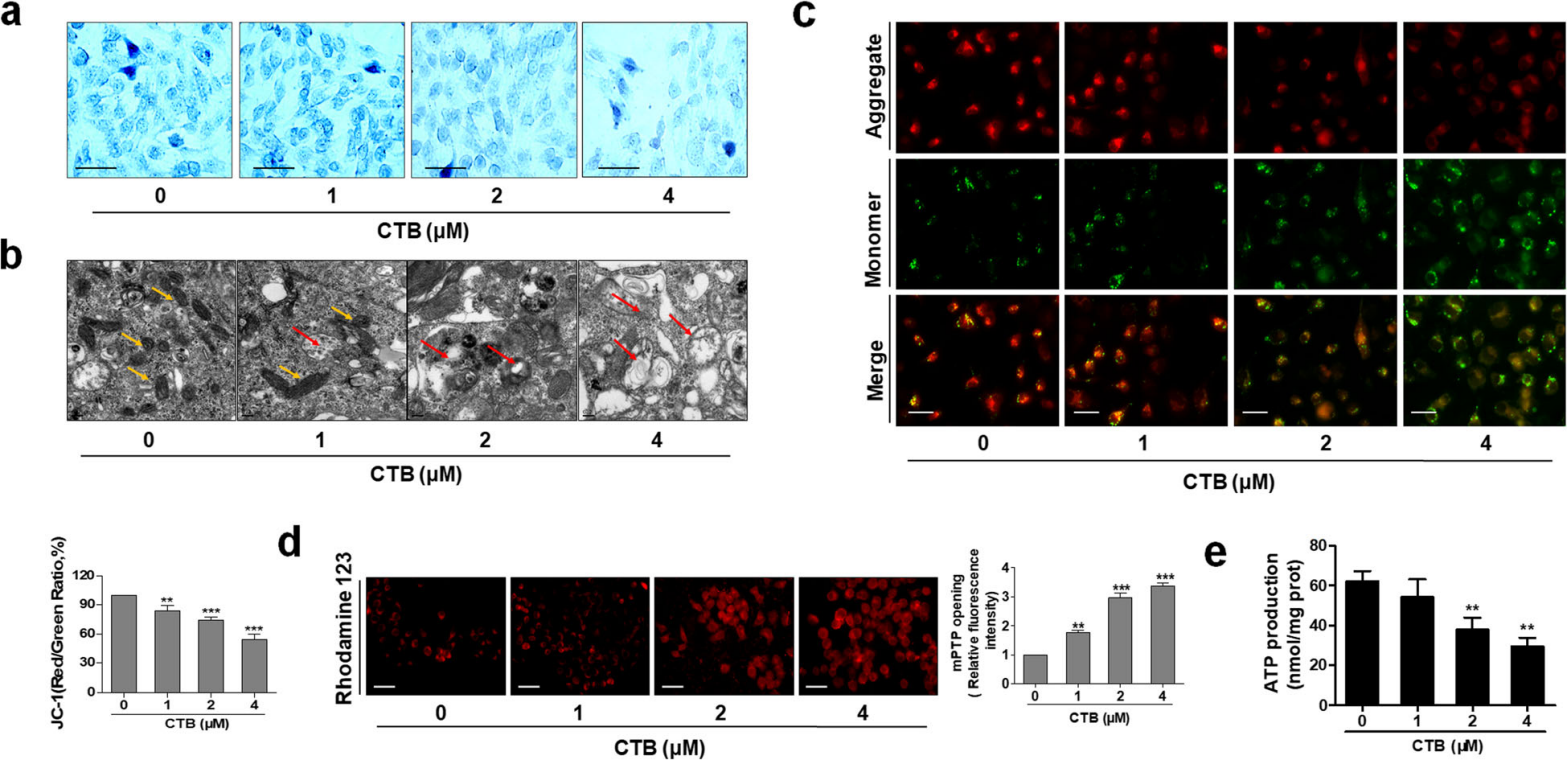
CTB destroyed mitochondrial structure and function of hepatoma cells. SMMC-7721 cells were treated with different CTB concentrations (0, 1, 2, 4 μM) for 24 h. **a** Janus green staining for detecting mitochondrial activity. Representative photographs were shown. Scale bar: 50 μm **b** Observation of mitochondrial morphology by Transmission Electron Microscope (TEM). Scale bar: 0.2 μm. **c** Effect of CTB on MMP (ΔΨm) was determined in SMMC-7721 cells by JC-1 staining. Scale bar: 50 μm. **d** The opening of mPTP was detected by Rhodamine123 staining. Scale bar: 50 μm. **e** ATP generation was detected by ATP Detection Kit. Scale bar: 50 μm. Scale bar: 50 μm. Data are represented as the mean ± SD (*n* = 3). *P < 0.05, **P < 0.01 and ***P < 0.001 vs Control

More deeply, Janus green staining showed that CTB destroyed the mitochondrial basic morphology and mitochondrial vigor in a dose-dependent manner (Fig. 2a). We further directly observed the effect of CTB on mitochondrial morphology and structure in hepatoma cells by using electron microscopic analysis. SMMC-7721 cells also showed typical lesion morphological changes, mitochondrial swelling, mitochondrial outer membrane rupture, and mitochondrial disorder (yellow arrow indicating healthy mitochondria; red arrow indicating damaged mitochondria) (Fig. 2b). Moreover, mitochondrial ΔΨm-sensitive dye JC-1 was used to detect the MMP in SMMC-7721cells treated with CTB. The high ΔΨm of control cells loaded with JC-1 allows for the formation of red-fluorescent J-aggregates. Upon loss of ΔΨm, these J-aggregates dissipate into monomers leading to a shift from red to green fluorescence. The dose-dependently decreased in red fluorescence and increased in green fluorescence were observed, indicating the dissipation of MMP (Fig. 2c). Rhodamine 123 fluorescence images also showed that CTB promoted the opening of mPTP to a certain extent (Fig. 2d). At the same time, there was a significant decrease in intracellular ATP levels, showing that CTB could influence the supply of cellular ATP to regulate cellular energy homeostasis (Fig. 2e). In summary, as a novel mitochondrion-targeting copper complex, CTB can exert good antitumor activity in liver cancer cells.

### CTB triggers apoptosis in hepatoma cells via the mitochondrial-mediated intrinsic pathway

**Fig. 3:**
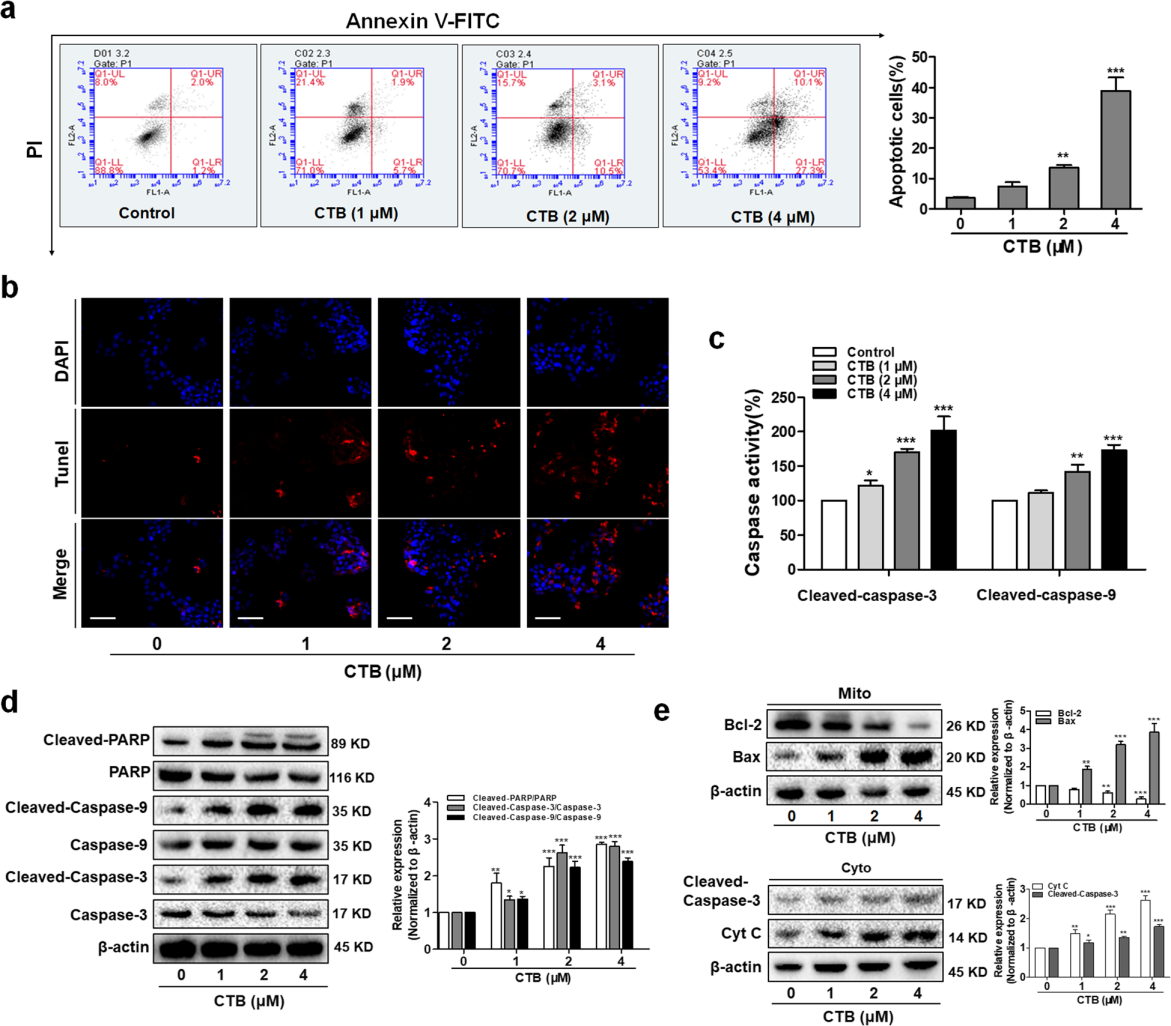
CTB triggered apoptosis through the mitochondrial death pathway in SMMC-7721 cells. Cells were treated with CTB at the indicated concentrations (0, 1, 2, 4 μΜ) for 24 h. **a** Flow cytometry analyses of cells apoptosis using FITC-labeled Annexin-V/PI staining. Cells situated in the right two quadrants of each plot were regarded as apoptotic cells. **b** TUNEL staining evaluated cells apoptosis. Red fluorescence indicated apoptotic cells. Scale bar: 50 μm. **c** Activity of caspase-3/9 was evaluated by caspase colorimetric assays. **d** The expression of Caspase cascades and PARP were determined by Western Blot. **e** The expression of Bcl-2/Bax and the release of Cyt C were determined by Western Blot. Data are represented as the mean ± SD (*n* = 3). *P < 0.05, **P < 0.01 and ***P < 0.001 vs Control

It was well known that the opening of mPTP was an important event in cell apoptosis. Once the loss of ΔΨm and opening of mPTP, some apoptosis-related molecules were able to flow across the mitochondria uncontrolledly. To further determine whether disruption of mitochondrial structure is accompanied with cell apoptosis, quantitative evaluation of apoptosis rate by flow cytometry showed that CTB induced SMMC-7721 cells apoptosis in a dose-dependent manner (Fig. 3a). When genomic DNA is cleaved, the exposed 3′-OH can be catalyzed by terminal deoxynucleotidyl transferase (TdT) plus fluorescein (FITC)-labeled dUTP (fluorescein-dUTP), so TUNEL (TdT-mediated dUTP Nick-End Labeling) staining could observe the occurrence of apoptosis (Fig. 3b). Caspases are produced as catalytically inactive zymogens in cells and must undergo proteolytic activation during apoptosis [35]. Consistent with these findings, the same CTB concentrations and exposure intervals resulted in cleavage/activation of caspase-3/9 and degradation of PARP (Fig. 3c, d). On this basis, we detected the release of apoptogenic factors in the cytoplasm, which further executed the caspase cascade pathway. Western blot analysis showed that treatment of cells with CTB resulted in a dose-dependent increase in Cyt C release from mitochondria into the cytosol (Fig. 3e). The Bcl-2 family proteins are the best characterized regulators of apoptosis, including the anti-apoptotic members and pro-apoptotic members. Treatment of cells with CTB resulted in decrease in levels of Bcl-2 and increase in levels of Bax (Fig. 3e). Taken together, the results confirmed that CTB activated mitochondria-mediated apoptosis in SMMC-7721 cells.

### Reactive oxygen species (ROS) accumulation is involved in CTB-induced hepatoma cells apoptosis

**Fig. 4:**
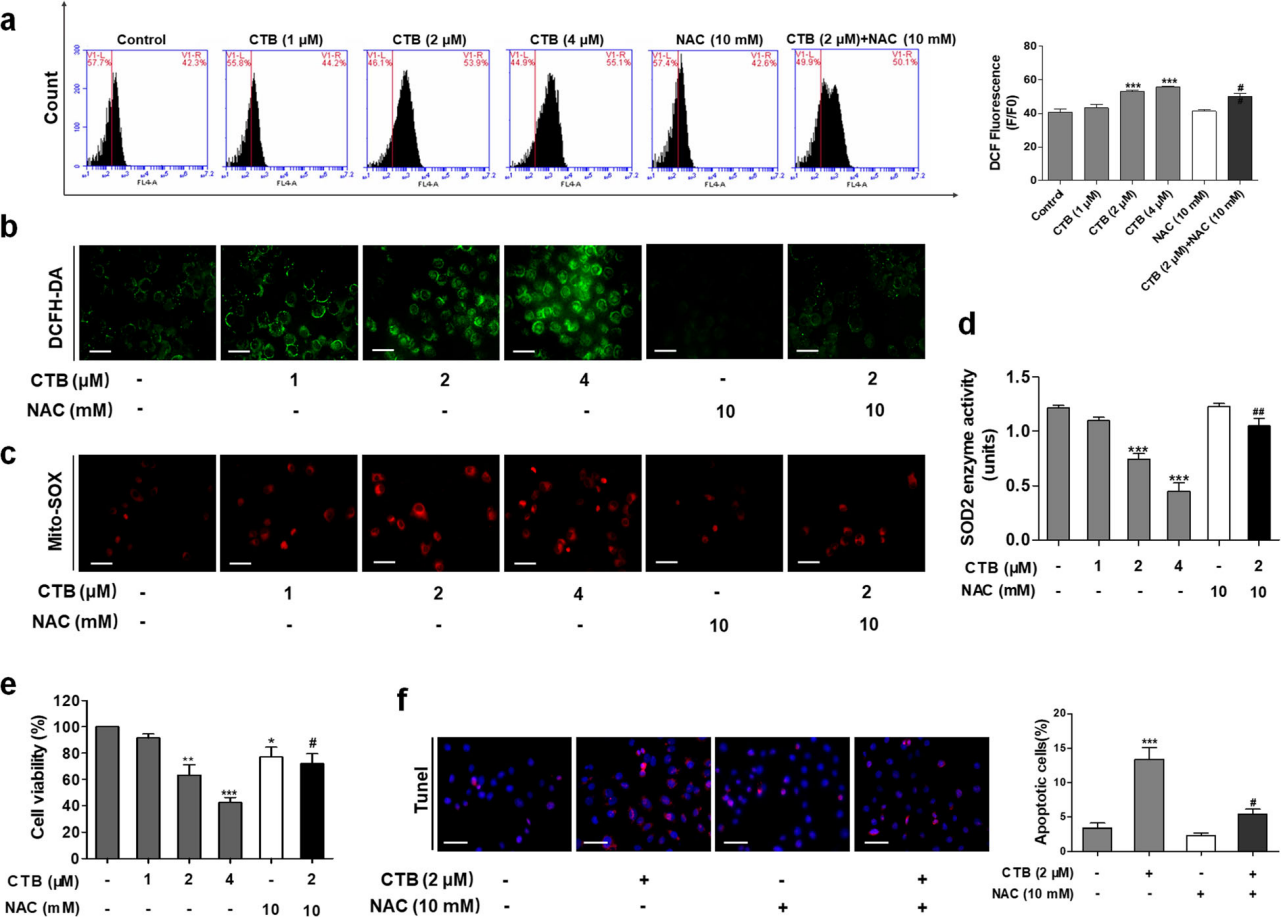
Reactive oxygen species (ROS) production were involved in CTB-induced hepatoma cells apoptosis. **a**, **b** Intracellular reactive oxygen species (ROS) was detected by Flow cytometry analysis and microscopic observation, using fluorescent probe DCFH-DA Scale bar: 50 μm. **c** MitoSOX Red immunofluorescence staining evaluated mitochondrial superoxide. Scale bar: 50 μm. **d** SOD2 enzyme activity was detected with Total Superoxide Dismutase Assay Kit with WST-8. **e** Cells were treated with CTB at the indicated concentrations (0, 1, 2, 4 μΜ) or NAC (10 mΜ) for 24 h. Cell viability was determined by the MTT assay. **f** TUNEL staining evaluated cells apoptosis. Data are represented as mean ± SD. Significance: *P < 0.05, **P < 0.01 and ***P < 0.001 vs Control; ^***#***^*P* < 0.05, ^***##***^*P* < 0.01 and ^***###***^*P* < 0.01 vs CTB (2 μΜ) treatment

In many cases, the cytotoxicity of copper complexes originates from the generation of ROS driven by the metal. Meanwhile, ROS plays a significant role in apoptosis activation by many chemotherapeutical drugs and radiation treatments [36, 37]. In the case of abnormal metabolism and signaling, tumor cells are well adapted to the unbalanced redox state through improved antioxidant capacity and oncogenic signaling pathways [22]. Due to its adaptive mechanism, cancer cells become insensitive even at high ROS levels. Importantly, we observed the loss of ΔΨm and the release of apoptotic factors after CTB treatment, allowing us to investigate the effect of ROS on CTB-induced mitochondria-mediated apoptosis. In combination, we used ROS scavenger, NAC (N-acetylcysteine). Initially, we used ROS-specific fluorescent probe DCFH-DA to determine ROS levels in SMMC-7721 cells. As shown in Fig. 4a and b, DCF fluorescence intensity (the oxidation of DCFH-DA by ROS) indicated that CTB significantly produced intracellular ROS in SMMC-7721 cells in indicated concentration using DCFH-DA staining and Flow cytometry. Treatment of NAC significantly scavenged the CTB-induced accumulation of intracellular ROS. We next investigated whether mitochondrial ROS production was also induced by CTB. Mito-Sox Red staining was used to evaluate the mitochondrial ROS production. As shown in Fig. 4c, CTB induced significant increase of mitochondrial superoxide, which was attenuated by NAC. The overproduction of ROS triggers serious damages in various cells, related to increased MDA levels and decreased SOD. We tested the level of mitochondrial SOD (also known as SOD2), and found that pretreatment of cells with administration of NAC significantly reversed CTB induced reduction of SOD2 levels (Fig. 4d). Importantly, CTB-induced apoptosis was greatly suppressed when cells were pretreated with the NAC (Fig. 4e-f). Collectively, these findings demonstrated that CTB-induced mitochondrial ROS production aggravated the occurrence of apoptosis in hepatoma cells.

### Activation of p53 and its mitochondrial translocation contribute to CTB-induced apoptosis

**Fig. 5:**
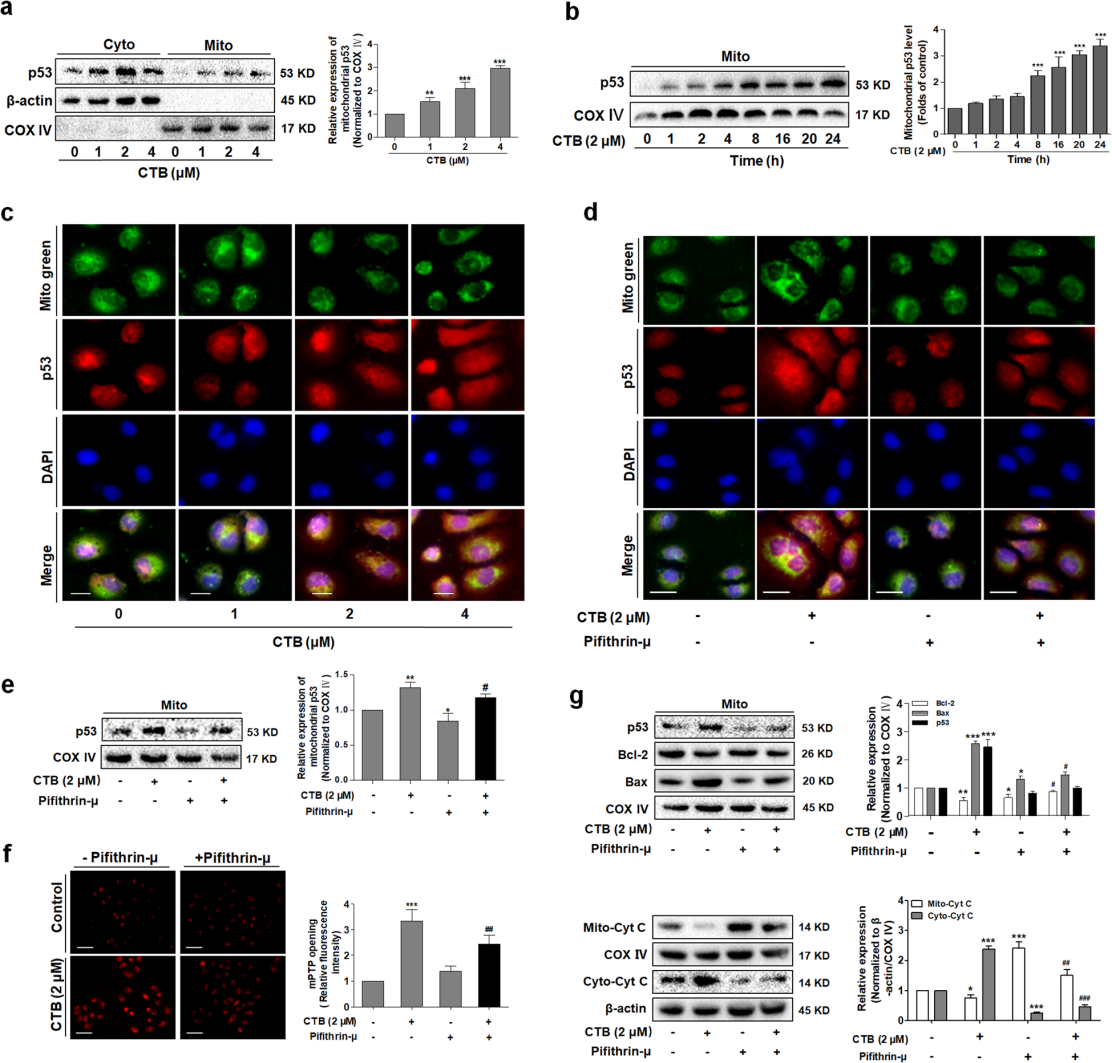
p53 is translocated into mitochondria in response to ROS, which contributes to CTB-induced apoptosis. **a** SMMC-7721 cells were treated with CTB at the indicated concentrations (0, 1, 2, 4 μΜ) for 24 h. Western blot analysis of p53 expression in cytoplasm or mitochondria respectively in SMMC-7721 cells. **b** Cells treated with CTB (2 μM) for the indicated time periods (1, 2, 4, 8, 16, 20, 24 h). Western blot analyses of p53 accumulation in mitochondria. **c** Representative Fluorescence microscope imaging of SMMC-7721 cells treated with CTB (2 μΜ) for 24 h, labeled with DAPI, anti-p53 antibody and Mito-tracker Green. Scale bar: 10 μm. **d** SMMC-7721 cells treated with the indicated concentrations of Pifthrin-μ (10 μM), CTB (2 μM) and Pifthrin-μ (10 μM) + CTB (2 μM) for 24 h. Representative Fluorescence microscope imaging of SMMC-7721 cells labeled with DAPI, anti-p53 antibody and Mito-tracker Green. Scale bar: 10 μm. **e** Western blot analyses of p53 expression in mitochondria. **f** The opening of mPTP was detected by Rhodamine123. Scale bar: 50 μm. **g** Western blot analysis of Bcl-2, Bax and p53 protein levels in mitochondria, and Cyt C release from mitochondria to cytoplasm. Data are represented as mean ± SD. Significance: *P < 0.05, **P < 0.01 and ***P < 0.001 vs Control; ^***#***^*P* < 0.05, ^***##***^*P* < 0.01 and ^***###***^*P* < 0.01 vs CTB (2 μΜ) treatment

Activation of p53 can occurs in response to a diverse number of cellular stress including DNA damage, cell death, oxidative stress and hypoxia [24]. Therefore, we want to further clarify whether CTB-induced apoptosis caused by ROS production is regulated by p53. p53 expression and subcellular distribution were determined. Compared with the cytoplasmic levels of p53, mitochondrial p53 showed a robust accumulation after SMMC-7721 cells exposure to CTB (Fig. 5a). Meanwhile, the level of mitochondrial p53 was also elevated in a time-dependent manner (Fig. 5b). Immunofluorescence staining further confirmed the above results. As illustrated by the increased fluorescent intensity and co-localization with Mito-tracker Green fluorescence, CTB significantly promoted the subcellular distribution of p53 (Fig. 5c). Further, mitochondrial distribution of p53 was reversed in the case of ROS clearance by NAC (Additional file 2: Figure S2A). NAC maintained the cytoplasmic levels of p53 and simultaneously inhibited mitochondrial levels of p53 by western blot analysis (Additional file 2: Figure S2B). The above results clearly indicated that in response to stress signal (especially ROS production), p53 would be transferred to mitochondria [38].

To further prove that mitochondrial translocation of p53 was a key step in activation of the mitochondrial apoptotic pathway induced by CTB in hepatoma cells, we treated SMMC-7721 cells with Pifthrin-μ, an inhibitor of p53 mitochondrial translocation. The decreased co-localization of p53 and mitochondria explained that Pifthrin-μ attenuated CTB-induced mitochondrial translocation of p53, observed by Immunofluorescence staining (Fig. 5d). Pifthrin-μ also reduced the level of mitochondrial p53 by Western blot analysis (Fig. 5e). The above results again demonstrated that CTB increased the level of mitochondrial p53. Through the detection of Rhodamine 123 staining in hepatoma cells, we found that Pifthrin-μ attenuated the opening of mPTP, suggesting that mitochondrial p53 could further aggravate mitochondrial damage (Fig. 5f). Simultaneously, concurrent with the use of Pifthrin-μ, the pro-apoptotic effect of CTB was attenuated and the regulation of Bax/Bcl-2 was weakened (Fig. 5g). Detection of the release of Cyt C from mitochondria to cytoplasm also confirmed that mitochondrial p53 promoted mitochondrial release of Cyt C to induce apoptosis (Fig. 5g). In addition, TUNEL staining also presented that CTB activated the p53 dependent pathway in mitochondria to induce the apoptosis (Additional file 2: Figure S2C). These results further validated that mitochondrial translocation of p53 played vital role in CTB-induced apoptosis of hepatoma cells.

In order to fully demonstrate that CTB-induced apoptosis of hepatoma cells is dependent on p53. Human hepatoma p53-null cells (Hep3B) and p53-mutant cells (Huh-7) were cultured for apoptosis analysis by flow cytometry. SMMC-7721 cells, Huh-7 cells and Hep3B cells were treated with CTB at 2 μΜ for 24 h. The results showed that the apoptosis rate of SMMC-7721 cells induced by CTB was higher than that of Huh-7 and Hep3B cells (Additional file 2: Figure S2D).

### Mitochondrial aggregation of Drp1 is involved in p53-induced apoptosis under conditions of oxidative stress

**Fig. 6:**
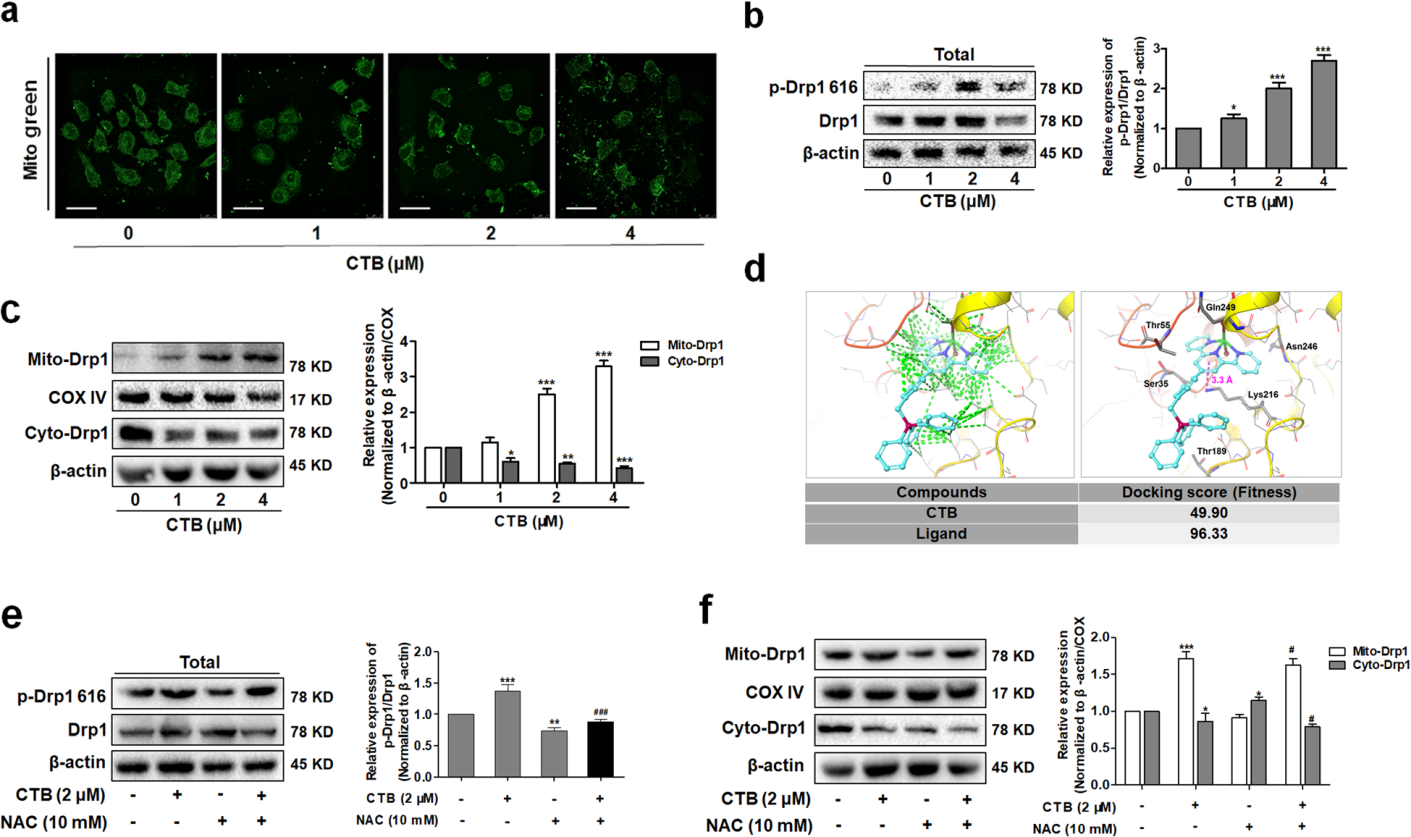
CTB promotes activation of Drp1 and mitochondrial aggregation by promoting ROS production. Cells were treated with CTB at the indicated concentrations (0, 1, 2, 4 μΜ) for 24 h. **a** Micrographs of mitochondrial morphology visualized by Mito-Tracker Green probe. Scale bar: 10 μm. **b** Western blot analysis of p-Drp1 (Ser616)/Drp1 expression. **c** Western blot analysis of Drp1 expression in cytoplasm or mitochondria respectively. **d** The molecular simulation used human Drp1 crystal (3W6P) as the acceptor, and the docking of CTB and 3W6P was detected by Gold docking software. The cyan small molecule represents the original ligand and the research molecule (CTB); the green dotted line indicated the intermolecular van der Waals force and the magenta dotted line indicated the positive Ion-Pi interaction. **e** Western blot analysis of p-Drp1 (Ser616)/Drp1 expression in SMMC-7721 cells treated with CTB (2 μM) and/or NAC (10 mM) for 24 h. **f** Western blot analysis of Drp1 expression in cytoplasm or mitochondria respectively. Data are represented as mean ± SD. Significance: *P < 0.05, **P < 0.01 and ***P < 0.001 vs Control; ^***#***^*P* < 0.05, ^***##***^*P* < 0.01 and ^***###***^*P* < 0.01 vs CTB (2 μΜ) treatment

Most mitochondria were filamentous and stable in hepatoma cells, whereas we observed that mitochondria were fragmented and massively accumulated in CTB-treated hepatoma cells, as assessed by fluorescence microscopy using Mito-tracker Green staining (Fig. 6a). Therefore, we deeply explored whether the effect of CTB on mitochondrial dynamics was involved in the induction of apoptosis. Then we identified key proteins regulating mitochondrial dynamics. CTB had no significant effect on mitochondrial fusion proteins, Mfn1 and Mfn2, which further cooperated with the occurrence of mitochondrial fission (Additional file 3: Figure S3A). However, CTB increased significantly the levels of p-Drp1 (Ser616) for activation of Drp1, the key protein regulating mitochondrial fission (Fig. 6b). Activated Drp1 gradually accumulated in the mitochondrial outer membrane to participate in the mitochondrial dynamics process [28]. As shown in the Fig. 6c and Additional file 3: Figure S3B, the protein level of Drp1 in mitochondrial fragments of hepatoma cells was gradually increased after CTB treatment in a time- and dose-dependent manner. These results indicated that CTB altered the balance of mitochondrial fission and fusion via regulation of Drp1 in hepatoma cells. Based on the above findings, we guessed whether CTB directly affect the activation of Drp1 in the cytoplasm, thereby exerting its mitochondrial biological activity. We used molecular simulation to explore our hypothesis that the original ligand of Drp1 and CTB were docked with the Drp1 crystal, respectively. The docking results showed that the chimerism of CTB and Drp1 was lower than the fit of Drp1 to the original ligand, and CTB had no direct effect on the phosphorylation-related sites (Fig. 6d). Based on the understanding of the effects of CTB on ROS, we speculated whether the production of ROS affected the activation of Drp1. Western blot analysis of p-Drp1 (Ser616) levels and mitochondrial Drp1 levels under ROS clearance, we found that NAC effectively inhibited Drp1 activation and mitochondrial translocation of drp1(Fig. 6e, f). These results indicated that under oxidative stress, CTB effectively induced the activation and translocation of Drp1, thereby interfering with the mitochondrial fission process. To prove the important role of Drp1-regulated mitochondrial fission in the process of CTB induced cell death, we used Mdivi-1 (specific suppression of Drp1) to analyze apoptosis. Both Immunofluorescence staining and Western blot analysis indicated that the expression of Drp1 was significantly inhibited by Mdivi-1 (Additional file 3: Figure S3C, D), and mitochondrial fission was weakened (Additional file 3: Figure S3E).

**Fig. 7:**
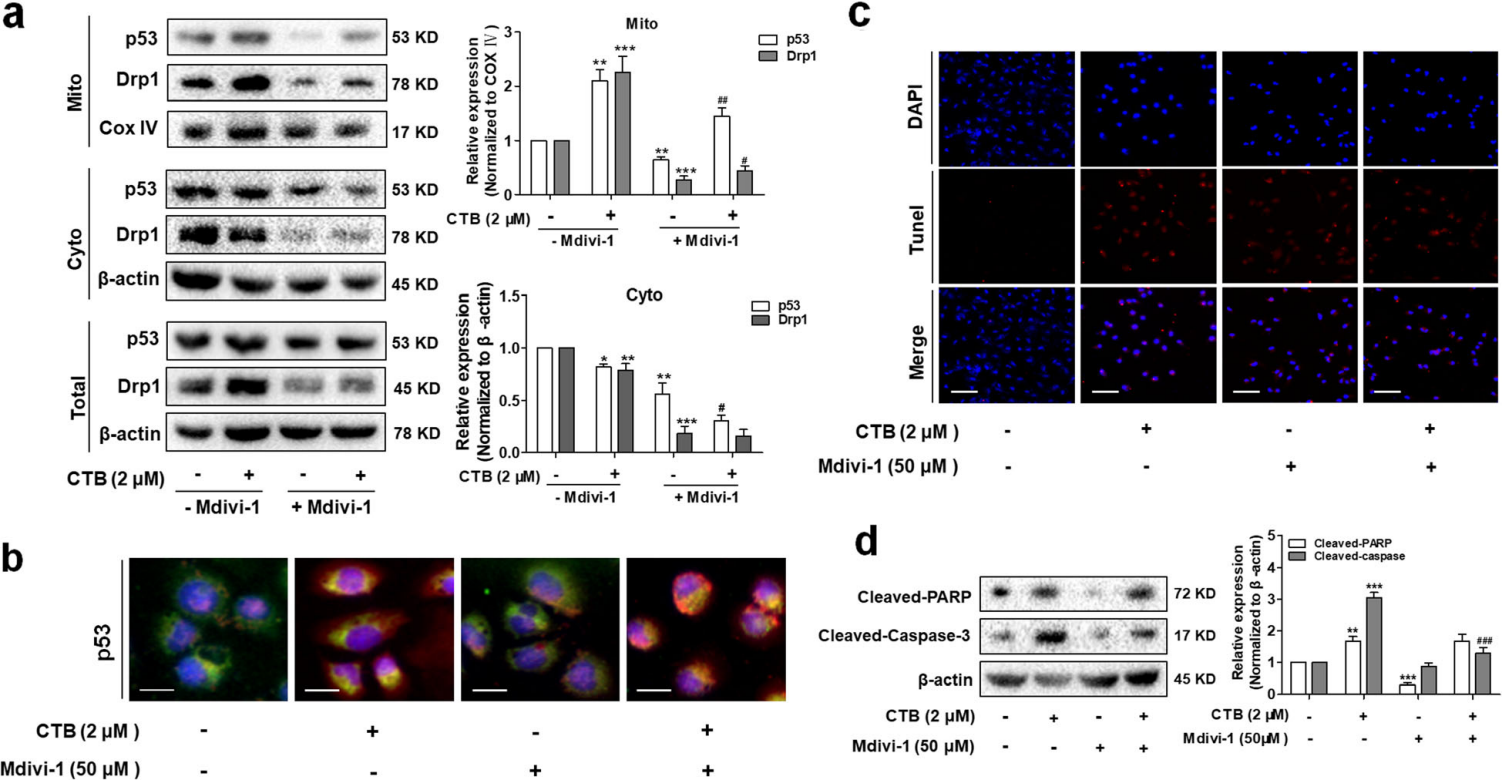
Activation of Drp1 is required for p53-dependent apoptosis **a** Mitochondrial translocation of Drp1 and p53 were detected by Western Blot. **b** Representative Fluorescence microscope imaging of SMMC-7721 cells labeled with DAPI, anti-p53 antibody and Mito-tracker Green. Scale bar: 20 μm. **c** TUNEL staining evaluated cells apoptosis. Red fluorescence indicated apoptotic cells. Scale bar: 50 μm. **d** Western blot analysis of cleaved-caspase-3 and cleaved-PARP levels in cytoplasm. Data are represented as mean ± SD. Significance: *P < 0.05, **P < 0.01 and ***P < 0.001 vs Control; ^***#***^*P* < 0.05, ^***##***^*P* < 0.01 and ^***###***^*P* < 0.01 vs CTB (2 μΜ) treatment

Once again, we detect the protein levels of Drp1 and p53 in mitochondria and cytoplasm respectively to explore whether mitochondrial translocation of p53 was dependent on Drp1. Drp1 knockdown markedly reduced the level of mitochondrial p53 induced by CTB compared to that in control cells (Fig. 7a). Co-localization of p53 and mitochondria also illustrated similar results by representative fluorescence microscope images of SMMC-7721 cells labeled with Mito-tracker Green (Fig. 7b). At the same time, we found that Mdivi-1 attenuated cells apoptosis induced by CTB, and inhibited the activation of PARP/casepase-3 in the cytoplasm, further indicating that Drp1-mediated mitochondrial fission was involved in cells apoptosis induced by CTB (Fig. 7c, d). Taken together, these discoveries showed that activation of Drp1 is required for p53-dependent apoptosis under conditions of oxidative stress.

### CTB inhibited tumor growth and induced cell apoptosis in vivo

**Fig. 8:**
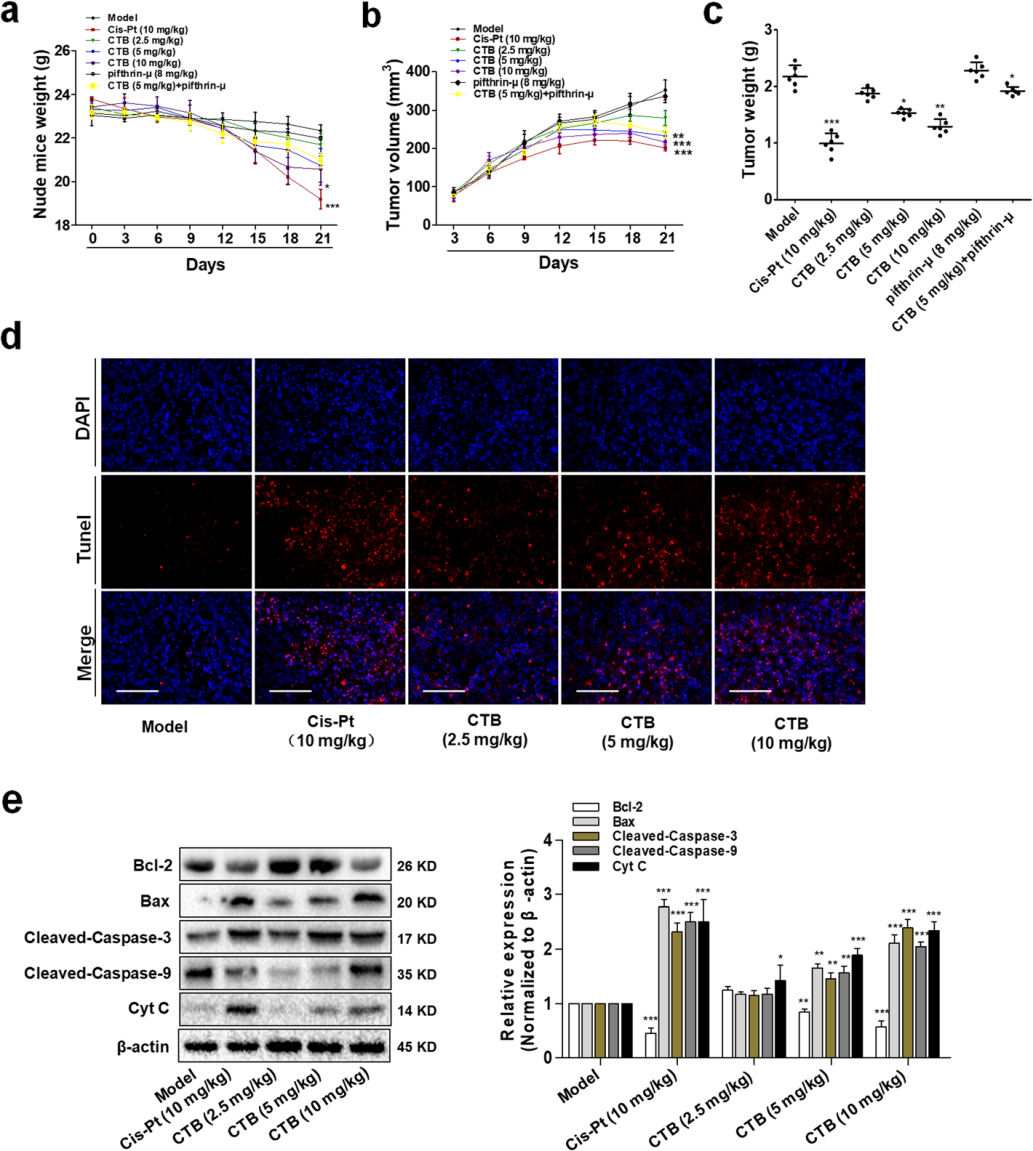
CTB inhibited tumor growth and induced cell apoptosis in vivo. BALB/c athymic nude mice inoculated with SMMC-7721 cells were treated with CTB (2.5, 5, 10 mg kg^− 1^), Cis-Pt (10 mg kg^− 1^) and vehicle solvent (0.9% saline solution) for 14 days. **a** The change of body weight in the CTB, Pifithrin-μ, CTB + Pifithrin-μ, Cis-Pt and vehicle-treated group (*n* = 6). **b** The growth curve of tumors, whose volumes were measured every 3 days in the CTB, Pifithrin-μ, CTB + Pifithrin-μ, Cis-Pt and vehicle-treated group (*n* = 6). **c** Tumor weights comparison, obtained on the final day of sacrifice in mice (*n* = 6). **d** Tunel staining of tumor tissue fixed in 4% paraformaldehyde (original magnification, 40×). Scale bar:100 μm. **e** Western blot analysis of cleaved caspase-3/9, Bax, Bcl-2 and Cyt C levels in tumor tissues. The data are represented as the mean ± SD (*n* = 6). *P < 0.05, **P < 0.01 and ***P < 0.001 compared with model group

To further assess the antitumor activity of CTB in vivo, we established human SMMC-7721 cells xenograft model, which was administered to CTB (2.5, 5 and 10 mg kg^− 1^), CTB (5 mg kg^− 1^) + Pifithrin-μ (8 mg kg^− 1^), Pifithrin-μ (8 mg kg^− 1^) and cisplatin (Cis-Pt, 10 mg kg^− 1^) for 14 days. In contrast, based on the mechanism of action of metal drugs on tumor DNA, the purpose of selecting cisplatin as a positive drug is to reveal whether the tumor suppressor rate of CTB exceeds cisplatin and has certain application prospects. Administration of drugs for 14 days, the tumor growth inhibition rate of CTB was 42.08%, which was slightly lower than that of Cis-Pt (Table 2). However, monitoring data on nude mice weight preliminarily indicated that the toxicity of CTB was weaker than that of cisplatin in vivo (Fig. 8a). Xenografts being treated with CTB had a markedly decreased growth rate in tumor volume as compared with model group (Fig. 8b, Additional file 4: Figure S4A). Consistently, the weight of control tumors was much higher than that of CTB-treated tumors (Fig. 8c). The above results indicated that CTB had the effect of inhibiting tumor growth. However, Pifthrin-μ weakened this effect (Fig. 8b, c).

**Table 2:**
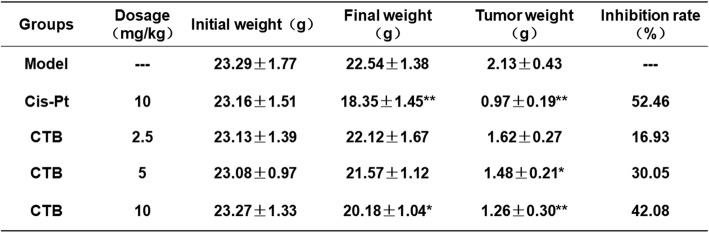
The inhibitory effect of CTB and cisplatin on xenografts, including initial body weight, terminal body weight and tumor weight in each group, and the tumor inhibition rate was calculated. *P < 0.05 and **P < 0.01 compared with model group

In the meantime, we also examined the role of CTB in inducing apoptosis in vivo. In HCC xenograf model, CTB promoted tumorigenic mitochondrial apoptosis in a dose-dependent manner, at a higher level than cisplatin (Fig. 8d, e). Combined with the cell culture experiment results, we more fully demonstrate that CTB has the ability to induce hepatoma cell apoptosis and the antitumor activity of CTB was further confirmed in vivo. The expression of p53 was detected by western blot after extracting tissue proteins cryopreserved in liquid nitrogen and separating mitochondrial proteins. Comparing the expression of p53 in cytoplasm and mitochondria, the results indicated that levels of mitochondrial p53 increased in tumor tissue (Additional file 4: Figure S4B). Immunofluorescence double staining images showed that mitochondria captured more p53 gradually, and the inhibitor could block this process (Additional file 4: Figure S4C). Combined with the cell culture experiment results, we more fully demonstrate that CTB has the ability to induce hepatoma cell apoptosis in vivo, which is accompanied by activation of mitochondrial p53.

## Discussion

The study of the antitumor mechanism of metal complexes mainly focuses on two aspects: (1) Act on DNA, which in turn blocks the cell cycle and induces apoptosis; (2) Act on enzymes (such as telomerase, topoisomerase, protein kinase, etc.), which affects the activity of tumor cells [39–41]. The difference in MMP between normal cells and tumor cells brings opportunity to design drugs that have preferential selectivity for tumor cells. In this study, we introduced the mitochondrial targeting group TPP into the copper-terpyridine complex to promote its cytostatic activity and selectivity to tumor cells. TPP is the most mature lipophilic cation and the most successful mitochondria-targeted ligand in current research, which contains three benzene rings and a delocalized positive charge so as to pass through the mitochondrial bilayer hydrophobic membrane [42]. Our previous research has demonstrated that CTB has the ability to inhibit the growth of cancer cells through multiple pathways, including the structural modification of DNA, scission of DNA strands, production of ROS, and dissipation of ΔΨm [16]. In the present study, we demonstrated that CTB potently induced mitochondrial injury and caspase-dependent apoptosis in human liver cancer cells. This phenomenon was primarily due to loss of MMP and opening of mPTP caused under CTB stimulation. mPTP opening has catastrophic consequences on the fate of cells, leading to release of Ca^2+^ from the mitochondrial matrix, ATP depletion, ROS production, and release of pro-apoptotic factors that trigger caspases activation and apoptosis [42]. Despite the potential for therapeutic development, molecular checkpoints responsible for mitochondrial apoptosis as a form of cell death remain poorly understood.

**Fig. 9:**
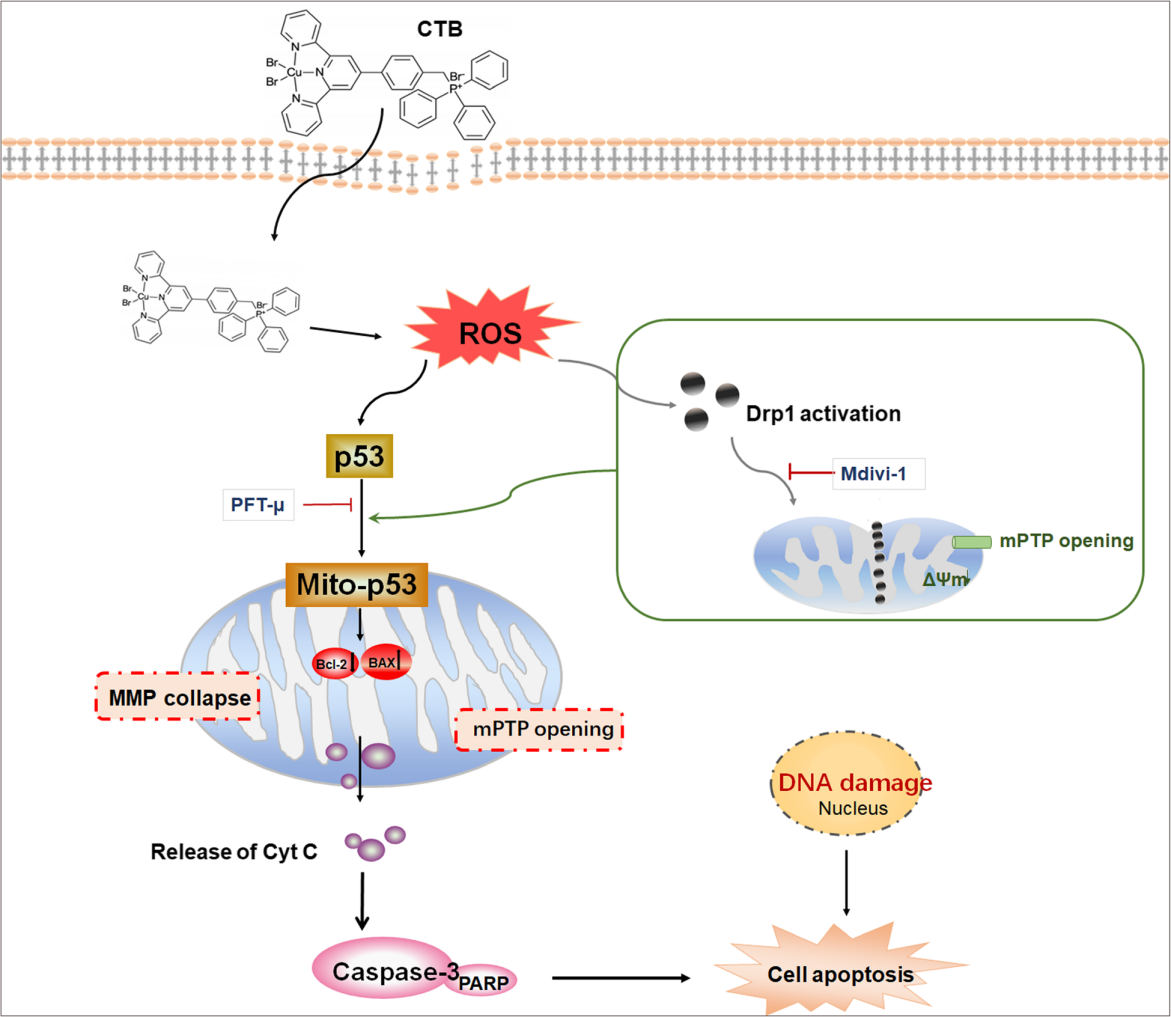
Illustration of the mechanism of CTB-induced apoptosis in SMMC-7721 cells. CTB induces excessive ROS production, and is able to facilitate ROS-mediated Drp1 activation, mitochondrial p53 accumulation, collapses of MMP, opening of mMTP and subsequent hepatoma cells apoptosis. Mitochondrial p53 is crucial for CTB-mediated cell apoptosis, which is associated with Bax mitochondrial recruiting and mitochondrial Cyt C releasing

Our results provided more specific information on the molecular mechanisms by which CTB induced mitochondrial apoptosis in hepatoma cells (i.e., by Oxidative stress, activation of Drp1, and mitochondrial translocation of p53, as shown in Fig. 9). ROS accumulation causes the oxidative stress that precedes cellular dysfunction and apoptosis, and is associated with the opening of mPTP [43]. The dissipation of the MMP leads to further ROS release from the mitochondria to the cytoplasm in a feedback loop [44]. CTB treatment induced the overproduction of intracellular ROS, thus explaining the ability of NAC to partially reverse the CTB-induced reduction in cell viability, ATP depletion, cell cycle arrest and effects on apoptosis. ROS has been reported to induce cell apoptosis by activating p53, but it is still unclear whether ROS-mediated activation of p53 has participated in CTB-induced apoptosis. Not only does p53 have a strong nuclear effect, its mitochondrial translocation may have a direct effect on mitochondrial function [45]. Therefore, we hypothesized that mitochondrial p53 may be involved in CTB-induced mitochondrial cell apoptosis under oxidative stress. To verify our speculation, we detected p53 expression in CTB and NAC treatment groups, and the results showed that CTB increased p53 expression levels in mitochondria under ROS accumulation. At the same time, the pifithrin-μ, a mitochondrial translocation of p53 inhibitor, inhibited p53 expression in mitochondria and cell apoptosis, which indicated that CTB increased cell apoptosis by promoting mitochondrial translocation of p53. The principle of action of pifithrin-μ is to reduce the affinity of p53 for the apoptotic protein Bcl-2, thereby inhibiting its binding to mitochondria. The results above suggested that p53 signal pathways in mitochondria were involved and played important roles in the process of CTB-induced, ROS-mediated cell apoptosis.

Cycles of fusion and fission mainly regulate the structure of mitochondria. The effect of mitochondrial fission on apoptosis has been confirmed by different laboratories in various cell models [28, 46]. Recently, Drp1-mediated mitochondrial fission plays a critical role in the regulation of cell cycle progression and hepatoma cell proliferation. Zhang et al. reported that mitochondrial fission promoted autophagy and apoptosis resistance in hepatoma cells through ROS-mediated coordinated regulation of NF-kappa B and p53 pathways [47]. Drp1 also interacts with Bax or Bak to disturb the MOMP, resulting in the release mitochondrial apoptotic proteins to trigger apoptosis [48]. In this study, the disruption of mitochondrial structure and imbalance between mitochondrial fission and fusion were observed in the CTB-treated hepatoma cells. These observations prompted us to explore the relationship between mitochondrial fission and CTB-induced mitochondrial apoptosis. Therefore, we suspected whether CTB could directly affect the biological activity of Drp1 and interfere with mitochondrial fission, involving in the process of apoptosis. This speculation was ruled out by molecular docking evidence showing that CTB could not bind to the Drp1 docking crystal. For further research, firstly, treatment of SMMC-7721 cells with NAC resulted in inactivation of Drp1 and decrease in mitochondrial Drp1 levels. A study has reported that the p53 up-regulated modulator of apoptosis (PUMA) was involved in Drp1 accumulation in mitochondrial membrane [49]. Yuan et al. reported that aldosterone-induced mitochondrial dysfunction and podocyte injury were mediated by p53/Drp1-dependent mitochondrial fission [50]. Meanwhile, pharmacological inhibition of Drp1 blocked mitochondrial fragmentation and mitochondrial release of cytochrome c and apoptosis [33]. Thus, we investigated the role of Drp1-dependent mitochondrial fission in p53-mediated mitochondrial apoptosis. Our results showed that pretreatment with Drp1 inhibitor Mdivi-1 suppressed mitochondrial fission, ROS-mediated mitochondrial translocation of p53, PARP degradation, and caspase 3 activation, as well as apoptosis. Taken together, our findings indicate that ROS, activation of Drp1 and mitochondrial p53 play crucial role in the regulation of mitochondrial dysfunction and mitochondrial apoptosis mediated by CTB.

Our research in vivo had shown that CTB markedly inhibited tumor growth in SMMC-7721 xenograft mouse model, and co-treatment with pifithrin-μ significantly weakened CTB-mediated suppression of tumor growth. These results suggested that sufficient CTB regimen can be achieved in vivo to recapitulate in vitro actions. However, we only obtained preliminary results in tumor-xenograft nude mice, which need further and deeper work for mitochondrial targeting research.

## Conclusions

We have fully demonstrated in vitro and in vivo that CTB exerts its anti-hepatocellular carcinoma effect by interfering with mitochondrial function and cell apoptosis. Once CTB enters the cell and disperses in the cytoplasm, it promotes ROS-mediated signal pathway, triggering the mitochondrial apoptotic pathway. We elucidated activation of Drp1 and mitochondrial p53 play crucial role in the regulation of mitochondrial dysfunction and mitochondrial apoptosis mediated by CTB. CTB has the capacity to selectively inhibit tumor cells with relatively low general toxicity, thus making us believe that copper complexes with mitochondrion-targeting potential can bring about unique anticancer efficacy unreachably by other metal drugs.

## Supplementary information

**Additional file 1:** **Figure S1.** Detection of toxicity of hepatoma cells and hepatocytes by CTB. (A, B) SMMC-7721 cells and LO2 cells were treated with CTB at different concentrations for 24 h, and the cell viability were quantified using the MTT assay. (C) LO2 were treated with CTB at different concentrations (0, 0.5, 1, 5, 10, 15, or 20 μM) for 24 h, and cell supernatant ALT/AST/LDH levels were detected by kits. Data are represented as the mean ± SD (*n* = 5). *P < 0.05, **P < 0.01 and ***P < 0.001 vs Control.

**Additional file 2:** **Figure S2.** CTB induced mitochondrial translocation of p53, which was closely related to ROS. (A) Representative Fluorescence microscope imaging of SMMC-7721 cells incubated with NAC (10 mM) or CTB (2 μM) for 24 h, labeled with DAPI, anti-p53 antibody and Mito-tracker Green. Scale bar: 10 μm. (B) Western blot analysis of p53 expression in cytoplasm or mitochondria respectively. (C) TUNEL staining evaluated cells apoptosis. (D) SMMC-7721 cells, Huh-7 cells and Hpe3B cells were treated with CTB at 2 μΜ for 24 h. Flow cytometry analyses of cells apoptosis using FITC-labeled Annexin-V/PI staining. Scale bar: 50 μm. Data are represented as mean ± SD. Data are represented as mean ± SD. Significance: *P < 0.05, **P < 0.01 and ***P < 0.001 vs Control; ^*#*^*P* < 0.05, ^*##*^*P* < 0.01 and ^*###*^*P* < 0.01 vs CTB (2 μΜ) treatment.

**Additional file 3:** **Figure S3.** Activation of Drp1 is required for p53-dependent apoptosis under conditions of oxidative stress. (**A**) Cells were treated with CTB at the indicated concentrations (0, 1, 2, 4 μΜ) for 24 h. Western blot detection of mitochondrial fusion protein Mfn1, Mfn2 expression. (**B**) Western blot detection of mitochondrial fission protein Drp1 expression. (**C**) SMMC-7721 cells treated with the indicated concentrations of Mdivi-1 (5 μM), CTB (2 μM), and Mdivi-1 (5 μM) + CTB (2 μM) for 24 h. Representative Fluorescence microscope imaging of SMMC-7721 cells labeled with DAPI and Drp1 antibody. Scale bar: 50 μm. (**D**) Western blot analysis of Drp1 expression in SMMC-7721 cell. (**E**) Micrographs of mitochondrial morphology visualized by MitoTracker Green. Scale bar: 10 μm. Data are represented as mean ± SD. Significance: *P < 0.05, **P < 0.01 and ***P < 0.001 vs Control; ^***#***^*P* < 0.05, ^***##***^*P* < 0.01 and ^***###***^*P* < 0.01 vs CTB (2 μM) treatment.

**Additional file 4:** **Figure S4.** CTB has the ability to induce hepatoma cell apoptosis in vivo, which is accompanied by activation of mitochondrial p53. (A) Photographs of tumors were separated from CTB, Cis-Pt and vehicle-treated group (Scale bar: 1 cm) (B) Western blot analyses of cytosolic and mitochondrial p53 protein levels. (C) Tumor sections were obtained, and p53 colocalization were viewed with fluorescence microscope (Blue: DAPI; Green: MitoTracker Green; Red: p53). Original magnification, 40×. Scale bar = 100 μm.

## Data Availability

The datasets used and/or analyzed during the current study are available from the corresponding author on reasonable request.

## Abbreviations

ALT: Alanine aminotransferase
AST: Aspartate aminotransferase
DAPI: Diamidino-phenyl-indole
DCFH-DA: 2,7-dichlorofluorescein diacetate
Drp1: Dynamin-related protein 1
HCC: Hepatocellular carcinoma
LDH: Lactate dehydrogenase
MMP: Mitochondrial membrane potential
MOMP: Mitochondrial out membrane permeabilization
mPTP: mitochondrial permeability transition pore
NAC: N-Acetyl-lcysteine
ROS: Reactive oxygen species
