# Supplementary figures and images for: TPP-related mitochondrial targeting copper (II) complex induces p53-dependent apoptosis in hepatoma cells through ROS-mediated activation of Drp1
Source: Cell Commun Signal. 2019 Nov 19;17:149. doi: 10.1186/s12964-019-0468-6 (PMC6862763; doi:10.1186/s12964-019-0468-6)

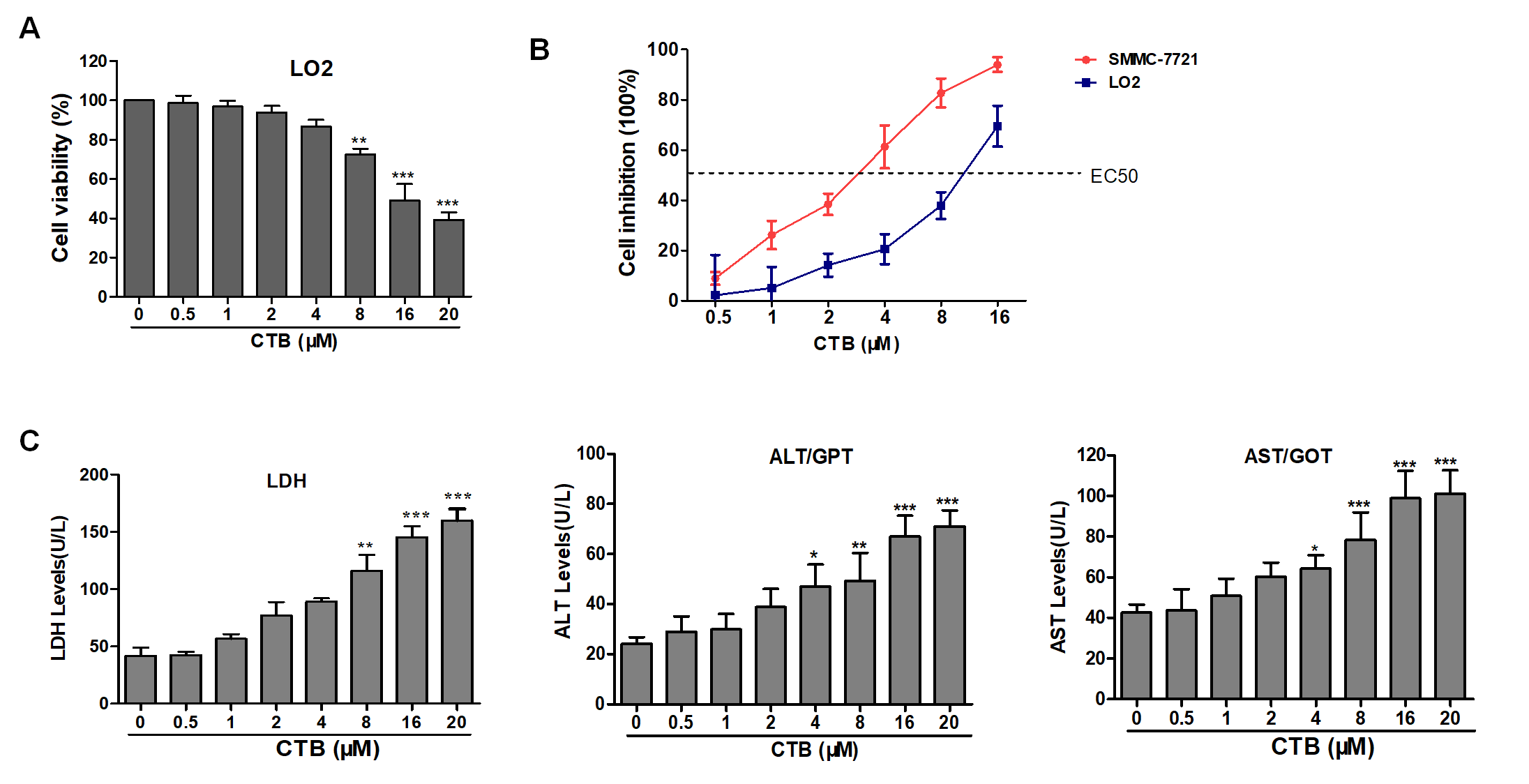

Supplement: Supplementary file 1 — Additional file 1: Figure S1. Detection of toxicity of hepatoma cells and hepatocytes by CTB. (A, B) SMMC-7721 cells and LO2 cells were treated with CTB at different concentrations for 24 h, and the cell viability were quantified using the MTT assay. (C) LO2 were treated with CTB at different concentrations (0, 0.5, 1, 5, 10, 15, or 20 μM) for 24 h, and cell supernatant ALT/AST/LDH levels were detected by kits. Data are represented as the mean ± SD (n = 5). *P < 0.05, **P < 0.01 and ***P < 0.001 vs Control. [file 12964_2019_468_MOESM1_ESM.tif]

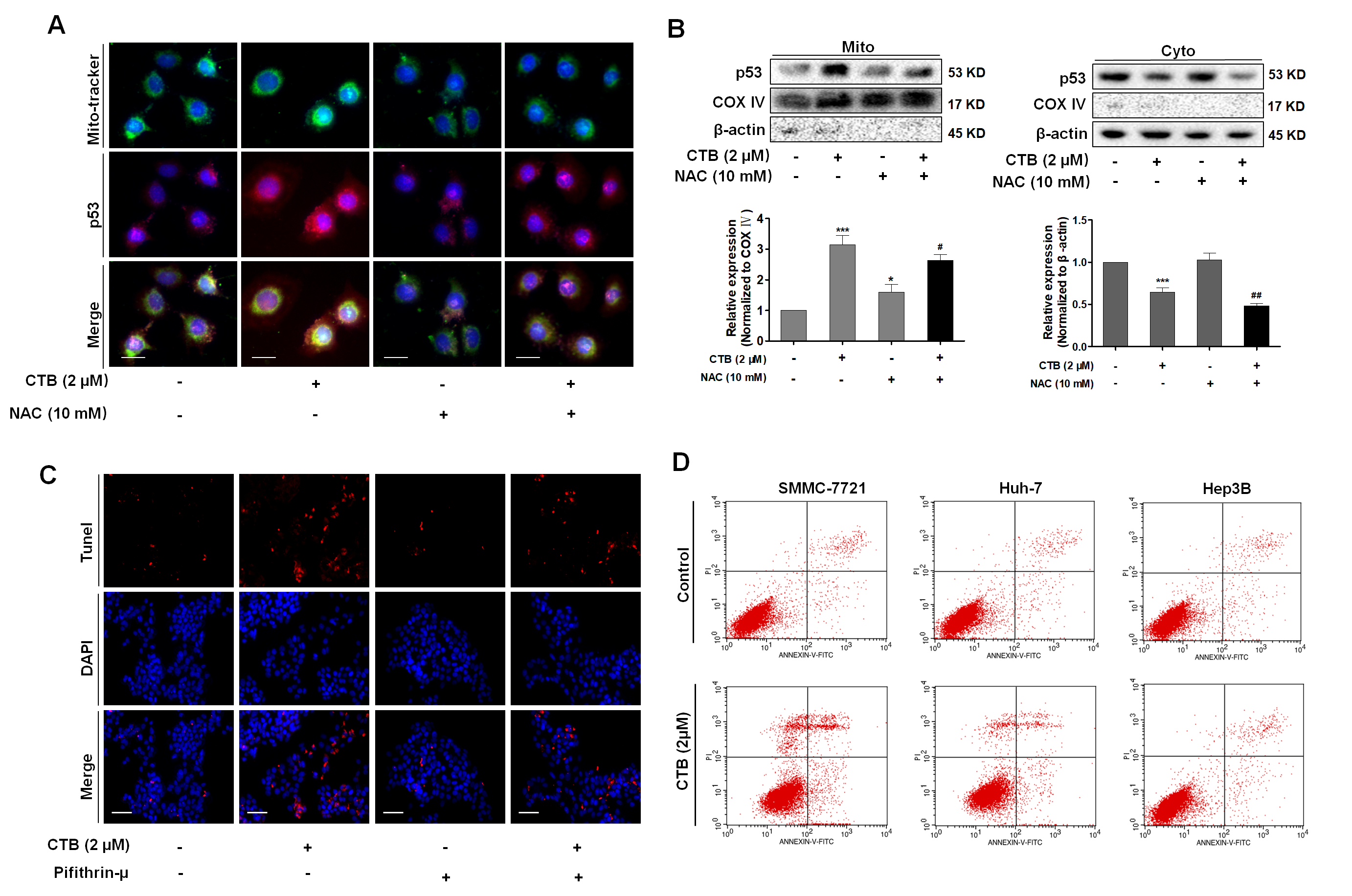

Supplement: Supplementary file 2 — Additional file 2: Figure S2. CTB induced mitochondrial translocation of p53, which was closely related to ROS. (A) Representative Fluorescence microscope imaging of SMMC-7721 cells incubated with NAC (10 mM) or CTB (2 μM) for 24 h, labeled with DAPI, anti-p53 antibody and Mito-tracker Green. Scale bar: 10 μm. (B) Western blot analysis of p53 expression in cytoplasm or mitochondria respectively. (C) TUNEL staining evaluated cells apoptosis. (D) SMMC-7721 cells, Huh-7 cells and Hpe3B cells were treated with CTB at 2 μΜ for 24 h. Flow cytometry analyses of cells apoptosis using FITC-labeled Annexin-V/PI staining. Scale bar: 50 μm. Data are represented as mean ± SD. Data are represented as mean ± SD. Significance: *P < 0.05, **P < 0.01 and ***P < 0.001 vs Control; #P < 0.05, ##P < 0.01 and ###P < 0.01 vs CTB (2 μΜ) treatment. [file 12964_2019_468_MOESM2_ESM.tif]

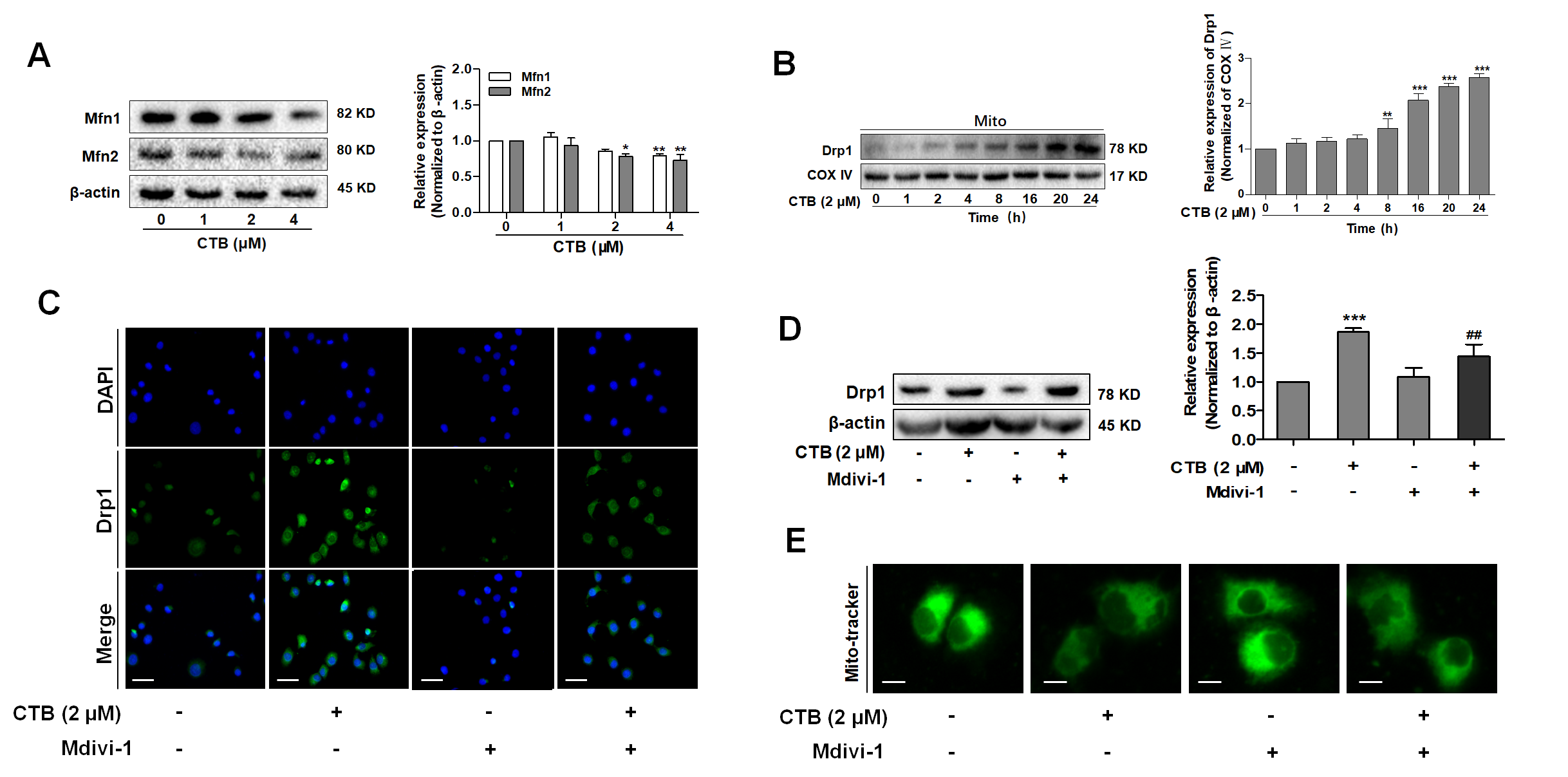

Supplement: Supplementary file 3 — Additional file 3: Figure S3. Activation of Drp1 is required for p53-dependent apoptosis under conditions of oxidative stress. (A) Cells were treated with CTB at the indicated concentrations (0, 1, 2, 4 μΜ) for 24 h. Western blot detection of mitochondrial fusion protein Mfn1, Mfn2 expression. (B) Western blot detection of mitochondrial fission protein Drp1 expression. (C) SMMC-7721 cells treated with the indicated concentrations of Mdivi-1 (5 μM), CTB (2 μM), and Mdivi-1 (5 μM) + CTB (2 μM) for 24 h. Representative Fluorescence microscope imaging of SMMC-7721 cells labeled with DAPI and Drp1 antibody. Scale bar: 50 μm. (D) Western blot analysis of Drp1 expression in SMMC-7721 cell. (E) Micrographs of mitochondrial morphology visualized by MitoTracker Green. Scale bar: 10 μm. Data are represented as mean ± SD. Significance: *P < 0.05, **P < 0.01 and ***P < 0.001 vs Control; #P < 0.05, ##P < 0.01 and ###P < 0.01 vs CTB (2 μM) treatment. [file 12964_2019_468_MOESM3_ESM.tif]

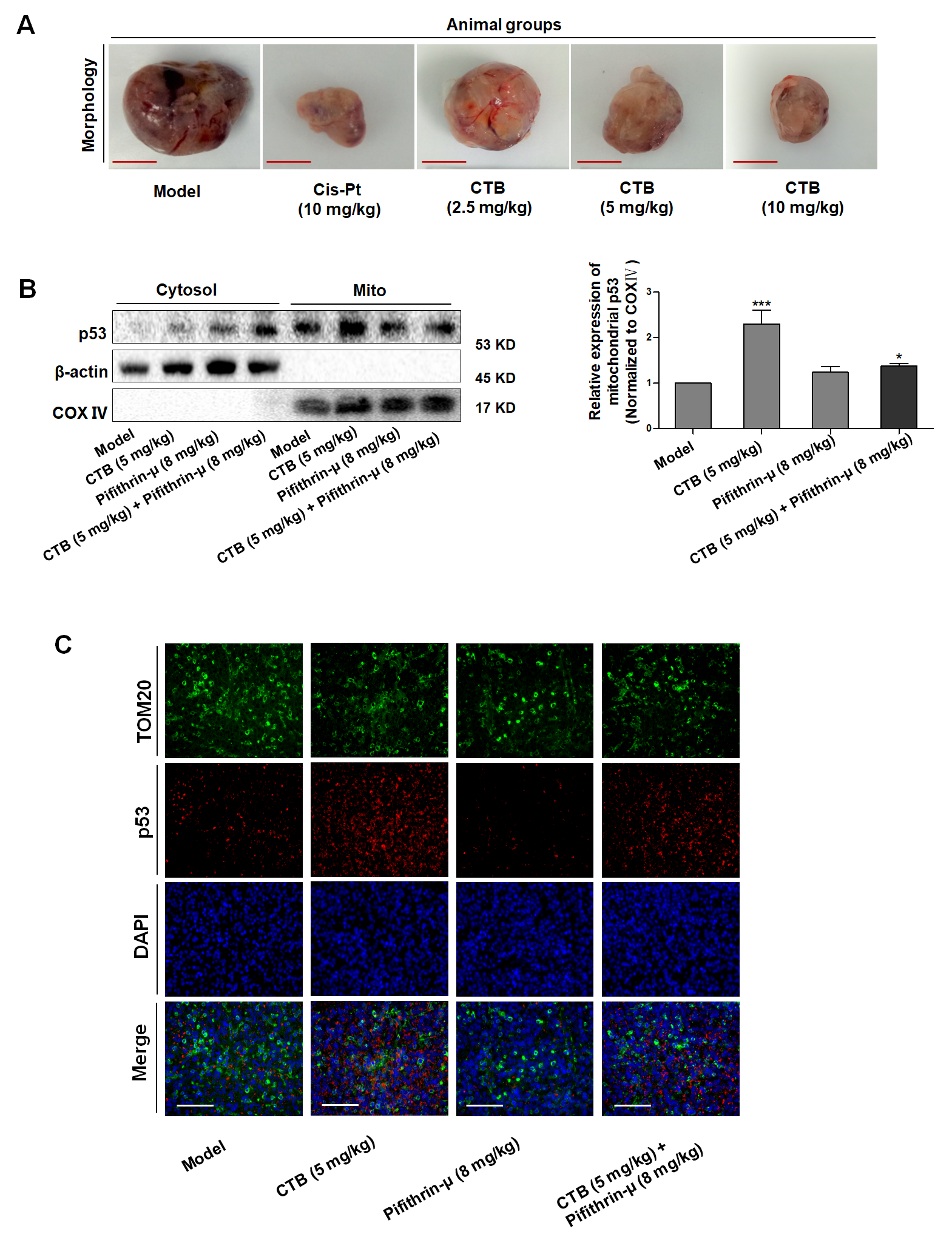

Supplement: Supplementary file 4 — Additional file 4: Figure S4. CTB has the ability to induce hepatoma cell apoptosis in vivo, which is accompanied by activation of mitochondrial p53. (A) Photographs of tumors were separated from CTB, Cis-Pt and vehicle-treated group (Scale bar: 1 cm) (B) Western blot analyses of cytosolic and mitochondrial p53 protein levels. (C) Tumor sections were obtained, and p53 colocalization were viewed with fluorescence microscope (Blue: DAPI; Green: MitoTracker Green; Red: p53). Original magnification, 40×. Scale bar = 100 μm. [file 12964_2019_468_MOESM4_ESM.tif]
